# The expression of long noncoding RNA NEAT1 is reduced in schizophrenia and modulates oligodendrocytes transcription

**DOI:** 10.1038/s41537-019-0071-2

**Published:** 2019-01-29

**Authors:** Pavel Katsel, Panos Roussos, Peter Fam, Sonia Khan, Weilun Tan, Tetsuro Hirose, Shinichi Nakagawa, Mikhail V. Pletnikov, Vahram Haroutunian

**Affiliations:** 1 Department of Psychiatry, The Icahn School of Medicine at Mount Sinai, New York, NY USA; 2 Department of Genetics and Genomic Sciences and Icahn Institute for Genomics and Multiscale Biology Friedman Brain Institute, The Icahn School of Medicine at Mount Sinai, New York, NY USA; 3 Mental Illness Research, Education and Clinical Center (MIRECC), James J Peters VA Medical Center, Bronx, NY USA; 4 Institute for Genetic Medicine, RNA Biology Laboratory, Hokkaido University, Sapporo, 060-0815 Japan; 5 RIKEN, RNA Biology Laboratory, Wako, Saitama, Japan; 6 Departments of Psychiatry, Neuroscience, Molecular and Comparative Pathobiology, Johns Hopkins University School of Medicine, Baltimore, MD USA; 7 Department of Neuroscience, The Icahn School of Medicine at Mount Sinai, New York, NY USA

## Abstract

Oligodendrocyte (OLG)-related abnormalities have been broadly observed in schizophrenia (SZ); however, the etiology of these abnormalities remains unknown. As SZ is broadly believed to be a developmental disorder, the etiology of the myelin abnormalities in SZ may be related to OLG fate specification during development. Noncoding RNAs (ncRNAs) are an important part of multifaceted transcriptional complexes participating in neurogenic commitment and regulation of postmitotic cell function. The long ncRNA, *NEAT1*, is a structural component of paraspeckles (subnuclear bodies in interchromatin regions) that may control activity of developmental enhancers of OLG fate specification. Gene expression studies of multiple cortical regions from individuals with SZ showed strong downregulation of *NEAT1* levels relative to controls. *NEAT1*-deficient mice show significant decreases in the numbers of OLG-lineage cells in the frontal cortex. To gain further insight into biological processes affected by *NEAT1* deficiency, we analyzed RNA-seq data from frontal cortex of *NEAT1*^*-/-*^ mice. Analyses of differentially expressed gene signature from *NEAT1*^-/-^ mice revealed a significant impact on processes related to OLG differentiation and RNA posttranscriptional modification with the underlying mechanisms involving Wnt signaling, cell contact interactions, and regulation of cholesterol/lipid metabolism. Additional studies revealed evidence of co-expression of *SOX10*, an OLG transcription factor, and *NEAT1*, and showed enrichment of OLG-specific transcripts in *NEAT1* purified chromatin isolates from human frontal cortex. Reduced nuclear retention of quaking isoform 5 in *NEAT1*^*-/-*^ mice shed light on possible mechanism(s) responsible for reduced expression of OLG/myelin proteins and supported the involvement of *NEAT1* in oligodendrocyte function.

## INTRODUCTION

Oligodendrocytes (OLG) dysfunction and myelin deficit are now well-established contributors to the pathophysiology of schizophrenia (SZ) (reviewed in ref. ^1^). The cause of broad range OLG-specific abnormalities in SZ remains unknown. Cell cycle abnormalities and incomplete differentiation of OLGs^2,3^ have been proposed as potential mechanisms that can impart reduced expression of the extensive list of OLG-specific genes seen in SZ. Whether these abnormalities are consequential to developmental or adulthood impairments, or to both, is uncertain.

The nuclei of higher eukaryotes are organized in subnuclear compartments^4^ and contain distinct subnuclear structures comprised groups of proteins and non-protein-coding RNAs (lncRNA, > 200 nt) that participate in specific nuclear processes.^5^ Many lncRNAs exhibit dynamic expression patterns during neuronal and glial cell lineage specification.^6^ *NEAT1*, nuclear-enriched abundant transcript 1 (aliases: TNCRNA, NCRNA00084), belongs to the group of lncRNA exhibiting highly abundant gene expression in the brain.^7,8^ Depletion of *NEAT1* leads to disintegration of paraspeckle^5^ subnuclear bodies, suggesting that *NEAT1* is a structural determinant of paraspeckles^9^ and serves as a scaffold for the bound core paraspeckle proteins: *PSPC1*, *FUS*, *NONO*, *TDP43*, and *SFPQ.*^9^ Two isoforms of *NEAT1* that share the same promoter are recognized, but differ in 3′-ends and length (3.7 and 23 kb in human and 3.2/ 20 kb in mice).^10^ *Neat1* and paraspeckles have been proposed to control stress responses,^11^ activation of innate immune responses,^12^ and cellular differentiation^13^ by sequestering RNA- and DNA-binding proteins,^11^ thus altering the epigenetic landscape of target gene promoters in favor of transcription.^14,15^ Changes in *NEAT1* expression have been found to be associated with development of multiple cancers^14,16^ and neurodegenerative diseases.^17,18^ These extensive regulatory functions of *NEAT1*-paraspeckle complexes together with its involvement in mRNA editing and retention^19^ provide mechanisms through which changes in *NEAT1* mRNA levels can strongly impact cellular function. Robust changes in *NEAT1* expression observed in mouse OLG precursors during differentiation suggested that *NEAT1* might dynamically modulate seminal fate decision within the OLG lineage.^20^

In the studies described below, we found dramatic downregulation of *NEAT1* in cerebrocortical regions of individuals with SZ compared with controls and show that *NEAT1* loss is associated with reduced populations of OLG-lineage cells and myelin-related gene expression changes in a NEAT1^-/-^ mouse model. These findings suggest a strong relationship between oligodendrogenesis/OLG-myelin gene expression and *NEAT1* expression, and that reduced *NEAT1* expression may be upstream of the oligodendroglial abnormalities observed in SZ.

## RESULTS

### NEAT1 is the top downregulated RNA transcript across multiple cortical regions in SZ

**Fig. 1:**
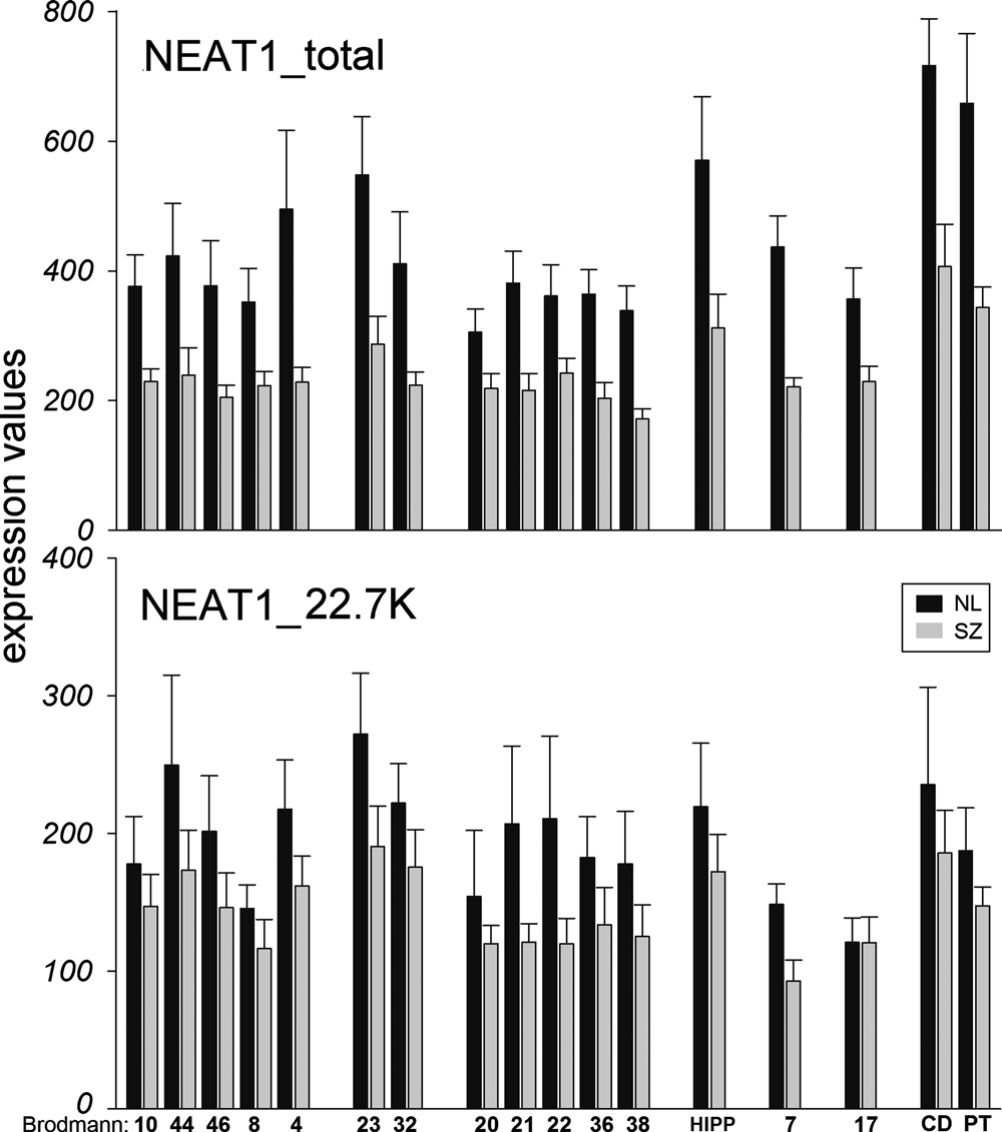
Gene expression changes of total *NEAT1* and long isoform *NEAT1_22.7* *K* in 17 brain regions in schizophrenia. Cortical Brodmann areas are marked. *HIPP* hippocampus, *CD* caudate, *PT* putamen. Data are expressed as mean ± SEM. Diagnosis effects are summarized in Tables S3 and S4

*NEAT1* sequences are represented by several probes on the HG-U133AB (Affymetrix) microarray chip recognizing both short and long variants of *NEAT1*. Analysis of variance for the expression of total (short + long) and the long variant of *NEAT1* (based on the probes: 224565_at and 225239_at, Table S2) derived from a previously described^21,22^ microarray study revealed a significant main effect of SZ diagnosis (F_1,506_ = 72.7, one-way analysis of variance (ANOVA); *p* = 1.7E − 16) for total *NEAT1* (short + long), and for the long variant (Table S2) of *NEAT1* (F_1,506_ = 21.7, one-way ANOVA; *p* = 4E − 06). Total *NEAT1* RNA levels were significantly decreased in all 14 cortical regions studied and, in the hippocampus (HIPP), caudate, and putamen of persons with SZ (Fig. 1, Table S3), whereas long*NEAT1* RNA levels were decreased only in the parietal cortex (BA7) of persons with SZ (Fig. 1, Table S3). However, when Brodmann areas were pooled on the basis of cortical lobe subdivision, the long*NEAT1* RNA levels were significantly decreased in frontal, temporal, and parietal cortices of individuals with SZ (Table S4). The total *NEAT1* mRNA level decrease in SZ was additionally validated in HIPP from an independent set of samples using quantitative PCR (qPCR) (F_1,45_ = 5.89, one-way ANOVA, *p* = 0.008, Fig S1). No significant correlations between potential covariates (age, pH, PMI, RIN, and gender) with total NEAT1 mRNA were detected in the full sample set. Although all donors with SZ had been exposed to antipsychotic medications for many years, a small subset (*N* = 5) in the qPCR study had been free of neuroleptic medication before death (for 4 weeks to 7 years; SZoff). No significant difference between SZ and SZoff groups was apparent (Student’s two-tailed *t*-test; *p* = 0.311, Fig S1), suggesting that the observed reduction of NEAT1 mRNA levels was independent of the acute effects of antipsychotic medications.

### Expression of Neat1 in OLG and effect of Neat1 knockout on OLG-lineage cells

**Fig. 2:**
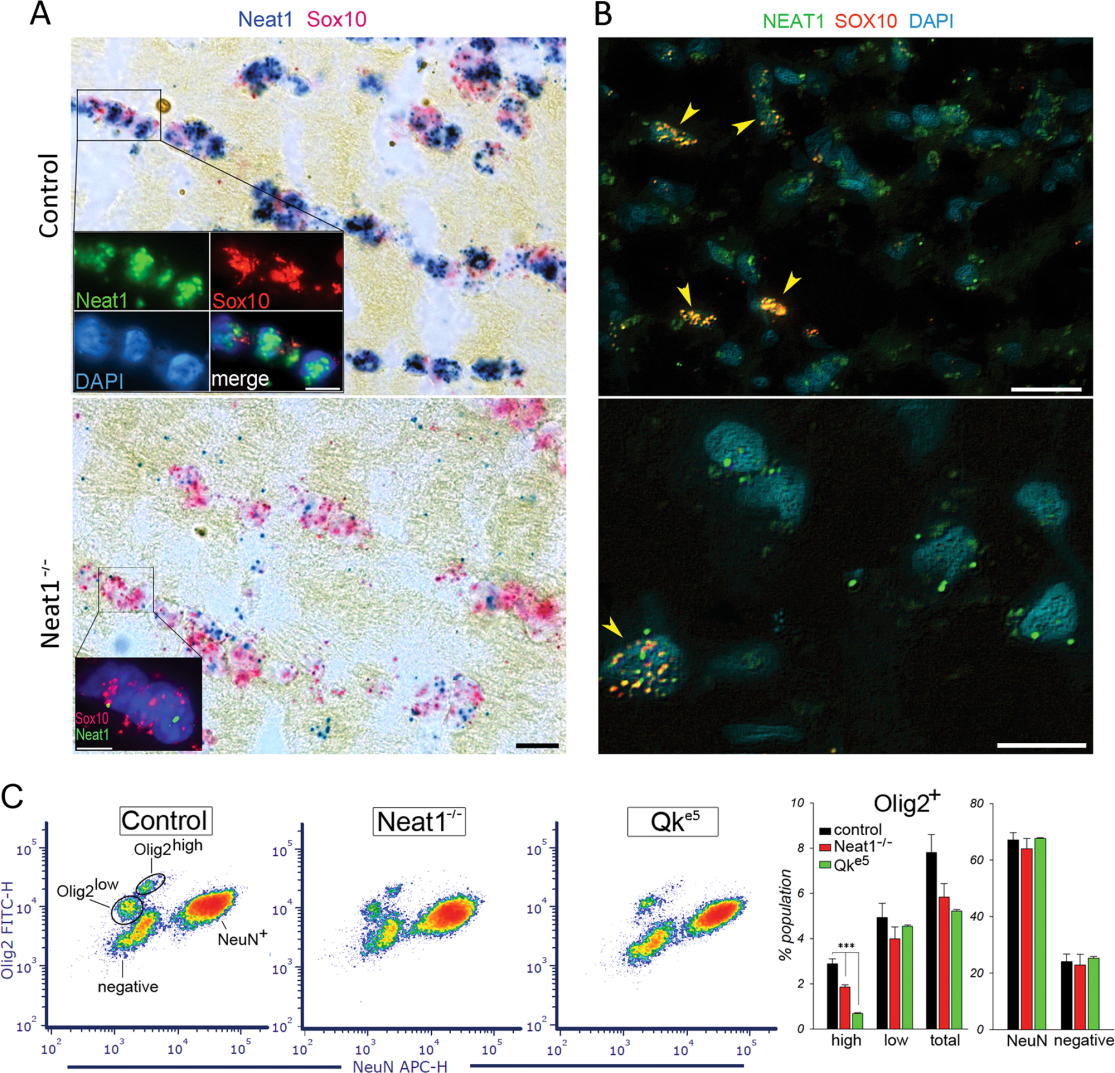
*Neat1* is expressed in OLGs and its knockout affects forebrain OLG-lineage cells. **a** Bright-field images of *Neat1* and *Sox10* ISH in murine striatum. Inserts from the frame: Fluorescent ISH for *Neat1* and *Sox10*. Cell nuclei were counterstained with DAPI. The scale bars, 10 µm. Lower panel: Neat1^-/-^ mice show depleted *Neat1* expression. **b** Fluorescent ISH for *NEAT1* and *SOX10* in human cingulate cortex (BA32). OLGs nuclei colocalization of *NEAT1* and *SOX10* is marked by arrowheads. Scale bars are 20 (upper) and 10 (lower) µm. **c** Flow cytometry scatter plots and analysis of *Olig2*^+^ and *NeuN*^+^ labeled cells from forebrains of controls, Neat1^-/-^, and Qk^e5^ mice. Data are expressed as mean ± SEM (*N* = 5/group). ***Student’s two-tailed *t*-test; *p* < 0.001

As *NEAT1* was present in OLG-specific gene clusters in the human microarray studies in SZ^22^ we performed in situ hybridization (ISH) of *Neat1* and *Sox10* (SRY-Box 10, OLG-lineage marker) in coronal sections of mouse and human brains. Strong expression of *Neat1* and *Sox10* was detected in nuclei of OLG-lineage cells in the frontal cortex and the striatum of control mice (Fig. 2a). The signal for *Neat1* (Fig. 2a) was almost abolished in the same regions of Neat1^-/-^ mice.^8^ ISH confirmed the presence of *NEAT1* in *SOX10*-positive nuclei of OLG-lineage cells in human cingulate cortex (Fig. 2b). To asses the effect of *Neat1* loss on OLG-lineage cells, we measured the numbers of OLGs (*Olig2*^+^) and neuronal (*NeuN*^+^) cell populations from frontal cortex of adult controls and Neat1^-/-^ mice using flow cytometry (Fig. 2c). Two well distinguished populations of OLGs were detected in controls representing OLG progenitors (*Olig2*^+high^) and mature OLGs *(Olig2*^+low^).^23^ Although *Olig2* (OLG-lineage transcription factor 2) is transiently expressed in developing neonatal astrocytes in the cerebral cortex, in adult mice *Olig2* is expressed exclusively in OLGs.^24^ A significant decrease of *Olig2*^+high^ cells (Student’s two-tailed *t*-test; *p*_s_ < 0.01) was detected in Neat1^-/-^mice, whereas the numbers of neurons (NeuN^+^), microglia, and astrocytes (*Olig2* and *NeuN* negative) were not significantly affected.

### Loss of lncRNA-Neat1 affects gene expression of ~1000 coding and noncoding RNAs in frontal cortex of Neat1^-/-^ knockout mice

**Fig. 3:**
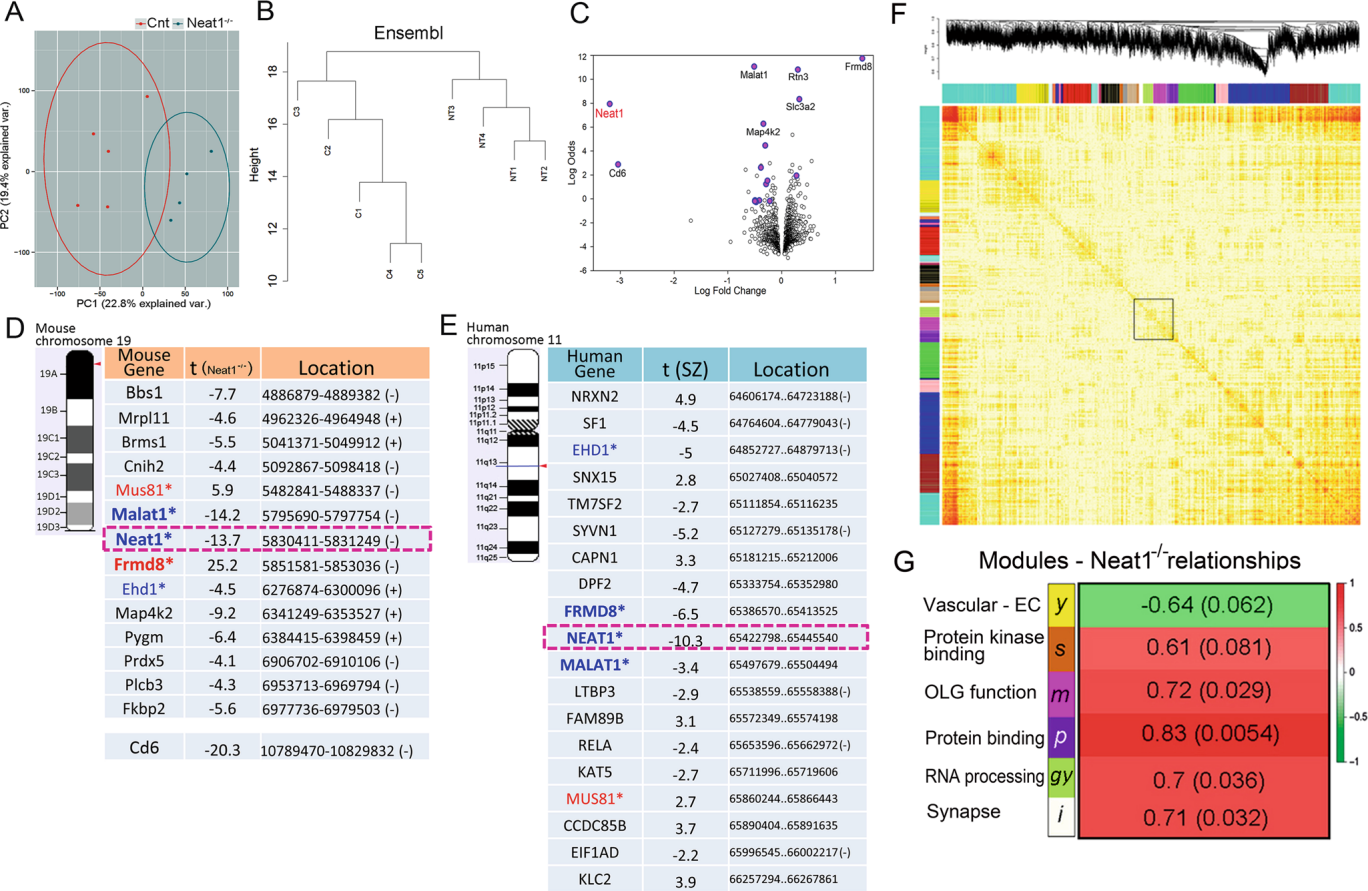
Differential gene expression analysis in frontal cortex of Neat1^-/-^ mice. **a** PCA of gene expression in frontal cortex between control (red) and Neat1^-/-^ (blue) mice. **b** Hierarchical cluster tree of RNA-seq samples shows separate clustering of samples from Neat1^-/-^ mice (*N* = 4) and controls (*N* = 5). **c** Volcano plot of mRNA expression. The DEGs located in Neat1 genomic region are marked. **d**, **e** DEGs in mouse (chr.19) and human (chr.11q13.1) genomic loci of *NEAT1* from Neat1^-/-^ mice and in SZ. Genes are organized based on the chromosome location, centered on *NEAT1*. Parentheses signs indicate DNA strand of transcription. Data are summarized in Tables S5–S6. Homologous genes are marked in bold (blue-downregulated; red-upregulated). **f** Hierarchical cluster tree of module’s eigengenes (top) co-expression network in murine frontal cortex and the topological overlap matrix (TOM) plot. The rows and columns represent the same set of genes sorted by the hierarchical clustering tree of TOM with modules represented by colored labels. The top affected modules highlighted by the rectangle. **g** Top affected modules associations with *Neat1* deletion. Numbers correspond to Pearson’s correlation (green to red gradient) and *p*-values (in brackets)

To study the downstream effect of *Neat1* RNA deficiency, we performed RNA-seq on frontal cortical specimens derived from adult 2–4 months old Neat1^-/-^ mice. Preliminary studies showed that these mice were viable and fertile under laboratory conditions, showing no evident grossly altered phenotypes except for the disappearance of paraspeckles.^8^ Both qPCR (Fig. S3) and RNA-seq showed near-complete depletion of *Neat1* RNA levels (logFC = −3.19, Student’s two-tailed *t*-test; *p* = 6.4E − 09, Fig. 3). Initial examination of RNA-seq data by principle component analysis (PCA, Fig. 3a) showed two principle components that were mutually uncorrelated and orthogonal. Hierarchical cluster tree analysis (Fig. 3b) showed that joined nodes from Neat1^-/-^ samples were distanced from control subset cluster confirming the results of the PCA. One thousand three hundred and fifty-nine genes were differentially expressed in the frontal cortex of Neat1^-/-^ mice (Fig. 3c). Twenty-five of these differentially expressed genes (DEGs) withstood Benjamini–Hochberg^25^ multiple testing corrections (fold change range 2–50). Four (including *Neat1*; > 1000 fold change) of the top five DEGs were adjacent to the *Neat1* locus (chromosome 19; Fig. 3c,d; Table S5) and include *Frmd8* (~30 fold), *Malat1* (~3-fold), and *Cd6* (>1000 fold). A positional gene enrichment analysis^26^ of human homologs of Neat1^-/-^ DEGs confirmed that the region centered around *NEAT1* on human chromosome 11 had the highest enrichment score (168.5; the probability of having the observed number of DEGs in the region was calculated by the hypergeometric distribution, adjusted *p*-val were calculated using the minimum *p*-val cumulative distribution function; *p* = 1.83E −14; Fig. S2). This prompted us to investigate genomic region proximal to the *Neat1* locus.

### Loss of Neat1 alters the expression profile of 21 transcripts proximal to its genomic locus

Further examination of the genes in the *Neat1* locus showed that 21 genes, positioned cis- and trans-, were significantly affected on mouse chromosome 19 and 19 of them within the ~3.9 Mb region centered around *Neat1* (Table S5 and Fig. 3c,d). The organization of the human and mouse *NEAT1* loci are similar. Nineteen genes were located within a 0.8 Mb region centered around *NEAT1* on human chromosome 11 (Table S6 and Fig. 3e). Five of these genes were *NEAT1*, *FRMD8*, *EHD1*, *MALAT1*, and *MUS81*, which are homologs of the mouse genes affected in Neat1^-/-^ frontal cortex. Analysis of the microarray dataset of 17 brain regions from individual with SZ showed that *NEAT1*, *FRMD8*, *EHD1*, and *MALAT1* were downregulated, whereas *MUS81* was upregulated in SZ, demonstrating directional similarity of the gene expression changes (except for the *FRMD8* mRNA levels) observed in SZ and Neat1^-/-^ mice, further corroborating possible association with downregulation of *NEAT1* in multiple brain regions in SZ.

### Functional enrichment of DEGs and gene co-expression networks in the frontal cortex of Neat1^-/-^ mice

We employed several annotation databases, including Metacore-Key pathway adviser, IPA, and ConsensusPathDB, to explore the neurobiological processes mediated by the DEG-associated pathways (1359 genes, Student’s two-tailed *t*-test; *p* < 0.05 without multiple testing correction). Summary of the key pathways and networks (Table S7) indicates that several processes related to OLG differentiation and myelination were affected in the frontal cortex of Neat1^-/-^ mice with the underlying mechanisms involving Wnt signaling, extracellular matrix and cell contacts interactions, and *VEGF/TGF* tyrosine kinase receptor signaling. Most, if not all, of these pathways evidence significant impact on the regulation of lipid and cholesterol metabolism pathways, which can significantly influence myelination. In addition, cholesterol and sterol biosynthesis-related pathways were identified through gene set enrichment analysis (GSEA, Table S9).

**Fig. 4:**
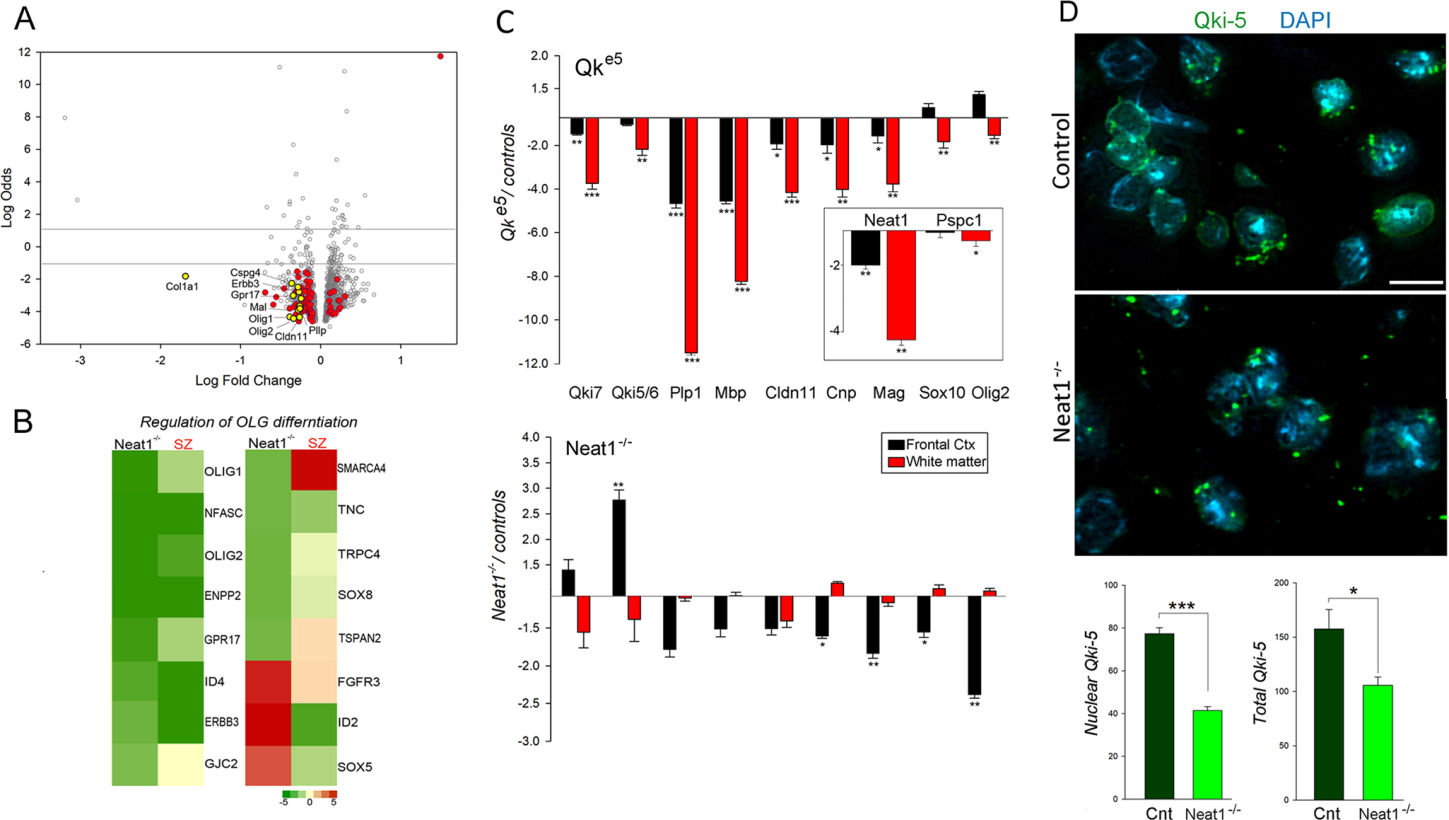
Neat1 expression is associated with OLG-specific markers. **a** Volcano plot of mRNA expression in frontal cortex of NEAT1^-/-^ compared with controls. The OLG-specific^66^ DEGs are highlighted (red) and labeled (yellow). **b** Heatmap showing gene expression of affected genes influencing OLG differentiation in Neat1^-/-^ and SZ. Scale indicates expression relative to their appropriate controls; *t*-scores and *p*-values are shown in Table 1. **c** Ratio plots (fold change ± SEM) of mRNA levels of OLG-specific genes, in the frontal cortex and white matter of Qki^e5^ and Neat1^-/-^ mice as determined by qPCR analyses (*n* = 6/group for Qki^e5^ and *n* = 5/group for Neat1^-/-^). Insert plot shows ratios for *Pspc1* and *Neat1* in Qki^e5^ mice. **d** Immunofluorescence staining of *Qki-5* in the forebrain of controls and Neat1^-/-^ mice. Cell nuclei were counterstained with DAPI. Scale bar, 10 µm. Immunostaining was repeated on nine sections from two independent animals from each experimental group. Fluorescence measurements in outlined DAPI-stained nuclei were performed using ImageJ 1.50b software (Wayne Rasband, NIH, USA) on six adjacent sections for each animal. Data are expressed as mean ± SEM. ***Student’s two-tailed *t*-test; *p* < 0.001; **p < 0.01; *p < 0.05

Next, we employed weighted gene co-expression network analysis (WGCNA) to capture the coordinated gene expression patterns associated with *Neat1* loss. The co-expression networks illustrated by the heat maps of topological overlap matrix plots show the top modules (Fig. 3f, marked by rectangle) that evidenced strong association with Neat1 deletion. Three out of four significant (Pearson correlation; *p* < 0.05) modules (Fig. 3g): purple, magenta and ivory modules, were significantly overrepresented by OLG-specific genes (8.5%; Fisher’s exact test; *p* = 8.02E − 05) compared with neural cell types and vascular endothelial cells (mean = 2.4%). GSEA indicated close relationships with reduced OLG-specific cellular function (oligodendrocyte_markers (70 genes); down; Student’s two-tailed *t*-test; *p* = 0.043; Table S9). Magenta module was enriched with OLG-specific DEGs (Fig. 4a) and biological processes associated with OLG/glial cell differentiation (Fisher’s exact test; *p* = 0.0017; Fig. 4b and Table 1), which included well-known regulators of OLG-lineage specialization, such as *Olig1*, *Olig 2*, *Gpr17*, *Sox5*, *Sox8*, *Smarca4*, *Id2*, and *Id4*; purple module, which included *Neat1*, was enriched with protein binding and response to abiotic stimulus; ivory module showed synaptic vesicle exocytosis, axon terminus, and neuregulin-ErbB signaling; and green yellow module was characterized by the nucleus and biological processes associated with RNA binding and processing (Fisher’s exact test; *p* = 7.06E − 04; Fig. S4, Table S8). Furthermore, the magenta OLG module was significantly enriched with *WNT* signaling cascade pathways (Fisher’s exact test; *p*_s_ ≤ 0.003), including Wnt-mediated activation of dishevelled (Dvl).

**Table 1:** OLG differentiation category (magenta module; Fig. 4b) affected genes in frontal cortex of Neat1^-/-^ mice identified by IPA

Symbol	Gene name	*Neat1* ^*-/-*^ *t*_s_	*p*-val	*SZ t*_s_	*p-*val^a^
*OLIG1*	Oligodendrocyte transcription factor 1	−4.79	4.2E − 04	−1.29	NS
*NFASC*	Neurofascin	−4.34	9.4E−04	−8.12	8.9E − 13
*OLIG2*	Oligodendrocyte transcription factor 2	−4.27	0.001	−2.86	6.1E − 03
*ENPP2*	Ectonucleotide pyrophosphatase/ phosphodiesterase 2	−3.41	0.005	−4.49	7.8E − 08
*GPR17*	G protein-coupled receptor 17	−3.16	0.008	−0.35	NS
*ID4*	Inhibitor of DNA binding 4, HLH protein	−2.74	0.018	−3.61	1.2E − 04
*ERBB3*	erb-b2 receptor tyrosine kinase 3	−2.36	0.036	−4.05	1.1E − 04
*TNC*	Tenascin C	−2.33	0.038	−1.97	NS
*TSPAN2*	Tetraspanin 2	−2.30	0.040	0.39	NS
*SOX8*	SRY-box 8	−2.30	0.040	−1.92	0.010
*SMARCA4*	SWI/SNF related, matrix associated, regulator of chromatin, subfamily a, member 4	−2.29	0.040	3.35	0.008
*TRPC4*	Transient receptor potential cation channel subfamily C member 4	−2.23	0.045	−1.76	NS
*GJC2*	Gap junction protein gamma 2	−2.11	0.050	0.55	NS
*SOX5*	SRY-box 5	2.28	0.041	−0.92	NS
*FGFR3*	Fibroblast growth factor receptor 3	2.98	0.011	0.45	NS
*ID2*	Inhibitor of DNA binding 2, HLH protein	5.18	2.2E − 04	−2.89	0.017

Corresponding *t*-scores across 17 cortical regions from individuals with SZ derived from microarray dataset (see Methods)

^a^SZ *p*-values are from moderated *t*-test with Benjamini and Hochberg multiple testing corrections. *NS* nonsignificant

### Chromatin purification with antisense NEAT1 probes showed enrichment of OLG-specific RNAs

Human postmortem frontal cortex chromatin isolation by RNA purification-sequencing (ChIRP-seq) RNA libraries were prepared from even and odd pools of the antisense *NEAT1* probes. Only transcripts identified in both RNA libraries were used. *NEAT1* was the most abundant RNA in both NEAT1 ChIRP-seq libraries. One thousand two hundred and thirty common transcripts were identified in both *NEAT1* libraries including mRNAs of three of the architectural core paraspeckles proteins (*NONO*, *PSPC1*, and *SFPQ*) that belong to *Drosophila* behavior/human splicing family.^27^ OLG-lineage cells specific transcripts (160 genes defined from Ref. ^23^; Table S10) were the most represented genes in ChIRP-seq compared with the other neural cell types (3-fold enriched over astrocyte; 1.5-fold over neurons, 20-fold over microglia, and 16-fold over endothelial cells). ChIRP-seq analyses showed presence of nodes of Ranvier structural proteins including neurofascin (*Nfasc)*, *opalin*, and contactin associated protein-like 2, *Cntnap2*/*Caspr2* (Table S10).

NEAT1-ChiRP also confirmed significant enrichment of genes involved in RNA processing (green yellow module, ratio = 0.75; Fisher’s exact test; *p* = 1.31E − 10, Table S8), OLG differentiation (magenta module, ratio = 0.77; Fisher’s exact test; *p* = 1.82E − 04; Table 1), and OLG-specific genes affected in SZ^2,22^ (Fisher’s exact test; *p* = 1.17E − 20). In addition, 16 of 19 gene transcripts (including *NEAT1*) within the human 11q13.1 genomic locus in proximity to *NEAT1* (Fig. 3e) were present in both ChIRP-seq libraries (Table S8) corroborating Neat1^-/-^ RNA-seq findings.

### Neat1 is associated with OLG/myelin-related gene expression signature in an independent myelin-associated mouse model

The genes affected in Neat1^-/-^ mice that were closely associated with OLG markers (Fig. 4a) and differentiation of OLG (magenta module (Fig. 4a, b)) showed similar reduction across the 14 accessed cerebrocortical regions in individuals with SZ, suggesting inhibition of OLG differentiation in SZ (Table 1). To explore the association of *Neat1* with OLG gene expression signatures further, we measured mRNA levels of *Neat1* and OLG-specific markers in the frontal cortex and white matter of demyelinating “quaking” Qk^e5^^28^ mice (Fig. 4c). QK^e5^ mice were selected for study, as quaking (Qki) is among the most affected mRNAs in the brains of persons with SZ^29,30^ and Qk^e5^ mice showed significant decrease of *Olig2*^+high^ cell population (Student’s two-tailed *t*-test; *p* < 0.001) in the forebrain similar to those observed in Neat1^-/-^ mice (Fig. 2c). *Neat1* gene expression was significantly decreased (Fig. 4c, insert) along with other OLG-specific markers in the frontal cortex and white matter of Qk^e5^ mice. Paraspeckles component 1 (*Pspc1*) mRNA levels were significantly decreased only in white matter of Qk^e5^ mice. Comparison of gene expression changes from frontal cortex of Neat1^-/-^ and control mice (Fig. 4c) showed significant (Student’s two-tailed *t*-test; *p*_s_ ≤ 0.05) reduction of OLG-specific genes (*Olig2*, *Cnp*, *Mag*, and *Sox10*) by qPCRs and validated by RNA-seq (Olig2, Mag, Cnp; Table 1 and S12). Importantly, the mRNA levels of these same OLG genes are among the most affected and replicated gene expression changes noted in SZ.^1,22,31^ Only two isoforms of quaking protein, *Qki* -5 and -6, showed significant (Student’s two-tailed *t*-test; *p* < 0.01) upregulation of mRNA levels in the frontal cortex of Neat1^-/-^ mice (Fig. 4c). Surprisingly, in contrast to the observations described above for gray matter, no significant changes were detected in the white matter of the Neat1^-/-^ mice (Fig. 4c), suggesting that additional factors may influence the impact of *Neat1* deletion on OLG-lineage cells in the white matter.

### Qki-5 nuclei retention is reduced in Neat1 KO mice

The *Qki* gene encodes several isoforms of *Qki* proteins, which belong to the STAR family of RNA-binding proteins and may be associated with paraspeckles and *Neat1*. We tested whether the loss of *Neat1* had an impact on nuclear retention of nuclear-specific RNA-binding protein, *Qki*-5 isoform.^32^ The nuclear and total signal of *Qki*-5 was significantly reduced (Student’s two-tailed *t*-test; *p* = 4.8E − 15; FC = − 1.86 for nuclear, and *p* = 0.027; FC = − 1.49 for total) in Neat1^-/-^ brains (Fig. 4d).

## DISCUSSION

The main findings of this study are as follows: (i) *NEAT1* is downregulated in multiple brain regions of individuals with SZ and is among the most affected genes in SZ; (ii) murine Neat1 deletion affected multiple genes involved in OLG cell differentiation accompanied by reduced population of OLG-lineage cells in the frontal cortex of Neat1^-/-^ mice; (iii) RNA-seq of human chromatin isolates purified with *NEAT1* antisense probes (ChiRP) identifies multiple OLG-specific RNAs, suggesting that *NEAT1* is an epigenetic regulator of OLG gene expression; (iv) *Neat1* loss is associated with reduced nuclear retention of the RNA-binding protein, Qki-5, and highlights additional mechanisms by which *NEAT1* may regulate OLG-specific differentiation.

Transcriptional signatures within magenta module of Neat1^-/-^ mice was enriched with genes participating in OLG differentiation and showed directional and functional similarity to the DEGs identified by multiregional microarray-based gene expression analysis in individuals with SZ. These modules and changes contrasted from changes in the same brain regions of individuals with Alzheimer’s disease (Fig. S5), indicating disease specificity and arguing against influence of neurodegeneration. These cross-validating observations support the overall hypothesis that *NEAT1* abnormalities in SZ may represent a core etiological component associated with the OLG/myelin abnormality linked to SZ.

Comprehensive review of OLG-specific genes differentially expressed in the frontal cortex of Neat1^-/-^ mice and human NEAT1 ChIRP-seq analysis showed the presence of genes encoding structural proteins of nodes of Ranvier including neurofascin, opalin, and contactin-associated protein-like 2. Notably, a recent postmortem study^33^ identified abnormalities in the expression of the structural proteins forming nodes of Ranvier in multiple cortical regions in individuals with SZ, emphasizing the contribution of OLG to the potential impairment of saltatory conduction and signal propagation in SZ. Moreover, neurofascin was a major disease-associated hub gene in OLG gene regulatory networks constructed from large-scale human postmortem gene expression data derived from persons with SZ.^34^

The *NEAT1* deficit in SZ may explain the cell cycle abnormalities that have been described in SZ.^2,35,36^ *Neat1* deletion in mice was associated with downregulation of well-known enhancers of OLG-lineage specialization, such as *Olig1* and *Olig2,*^37,38^ *Gpr17,*^39^ *Sox8,*^40,41^ and *Smarca4,*^42,43^ In addition, differential expression of inhibitors of DNA binding (*Id2* and *Id4*), the critical effectors of cell cycle transition, were detected in the frontal cortex of Neat1^-/-^ mice. Id2 interactions with retinoblastoma protein and bHLH transcription factors, such as *OLIG -1*, *-2*, and *E47*, in OLG precursor cells are thought to mediate inhibition of OLG differentiation,^44^ Our findings of upregulation of *Id2* in Neat1^-/-^ mice are consistent with in vitro studies showing that overexpression of *Id2* powerfully inhibits OLG differentiation, whereas the loss of *Id2* induces premature OLG differentiation.^45^ *Id4* is another member of the inhibitors of DNA binding family and has a critical role in the timing of OLG differentiation.^44,46,47^ Although overexpression of *Id4* inhibits OLG differentiation^47^ in a similar manner as *Id2*, the loss of *Id4* has been associated with reduced levels of several myelin-specific proteins.^46^ Additional factors may influence the impact of *Neat1* deletion on OLG-lineage cells as indicated by lack of similar changes of mRNA levels of selected myelin-specific genes in the white matter of the Neat1^-/-^ mice. As loss of Neat1 was not limited to the OLG-lineage cells, the crosstalk between regionally diverse cell types may contributed, at least in part, to OLG-related gene expression alterations.

Genes located in close proximity to the *Neat1* locus exhibited the strongest transcriptional dysregulation as a result of *Neat1* loss. *Malat1*, lncRNA situated only ~40 kb downstream of *Neat1* was among those genes. Similarly, the loss of the *Malat1* in adult mice affected many *Malat1* neighboring genes including *Neat1,*^48^ suggesting potentially mutual regulatory roles of *Neat1* and *Malat1* on the expression of genes on chromosome 19qA and the human 11q13.1 regions where both genes are colocalized. Of particular note, *MALAT1* mRNA levels were also significantly decreased in several cortical regions in SZ (Table S5). During activation of transcription, *Neat1* and *Malat1* exhibit colocalization to multiple genomic loci primarily over active genes, but display distinct gene structural binding patterns at these sites, suggesting independent but complementary functions for these lncRNAs.^15^ Transcriptional inhibition or stimulation alters localization of *Neat1* on active chromatin sites, indicating rapid and dynamic interactions with the cues involved in the gene transcription process.^15^ We confirmed high abundance of *MALAT1* in human chromatin isolates by *NEAT1* purification (Table S8) and showed enrichment of a large number of OLG-specific RNAs in chromatin-*NEAT1* complexes, suggesting additional ways in which NEAT1 and MALAT1 can modulate OLG transcription signature and gene-variants splicing, which needs to be explored further.

Recent studies have revealed that paraspeckles and *NEAT1* may target gene transcription by sequestration of RNA- and DNA-binding proteins.^11^ The studies described here show that nuclear retention of the KH domain RNA-binding protein—quaking 5 isoform, critical for OLG progenitors to mount cell cycle exit^49^ and initiation of the OLG cell-fate maturation sequence,^2,50–53^ was significantly decreased in frontal cortex of Neat1^-/-^ mice, providing clues about additional means by which *NEAT1* and paraspeckles may promote OLG differentiation and myelination.

Potential negative feedback of reduced nuclear retention of Qki-5 protein on *NEAT1* expression is also supported by the transcriptional changes in Qk^e5^ mice exhibiting demyelination^2^ in which *Neat1* was strongly downregulated in white matter along with several Qki isoforms and seven other myelin-related genes. That reduced *NEAT1* expression and Qk^e5^ share similar transcriptional profiles and potentially interact is particularly relevant in the context of SZ given they are among the most transcriptionally dysregulated genes in SZ. That they both have an impact on the expression of oligodendroglial genes suggest that they are of etiological relevance to SZ.

The downstream effects of *NEAT1* loss are not limited to OLGs lineage as its expression is abundant in other brain cell types, including neurons. The impact on neuronal phenotype is highlighted by the ivory module genes, which yielded terms closely related to synaptic vesicle exocytosis, axon terminus, and neuregulin-ErbB signaling. These alterations can be a result of impaired OLG/myelin function on myelinated axons, or the result of loss of endogenous neuronal *NEAT1*. A recent study examining the transcriptome of GABAergic and glutamatergic neurons in the human prefrontal cortex^54^ indicated that *NEAT1* is highly abundant in GABAergic interneurons, whereas nearly absent in glutamatergic projection neurons.^55^

Taken together, our results suggest involvement of *NEAT1* in the OLG function including myelination. Dysregulation of *NEAT1* has particular relevance to SZ, shedding light on the roles of lncRNAs in brain development, deepening and advancing our understanding of the mechanisms underlining the pathophysiology of SZ.

The present study has several limitations inherent to postmortem human brain research, including the absence of specimens from neuroleptic-naive individuals with SZ. These factors introduce considerable uncertainty into the analysis of potential medication effects. Although we did not find differences between individuals with SZ, who were free of neuroleptic treatment for extended periods of time before death, and the rest of SZ patients, we cannot completely exclude the effect of neuroleptic exposure on *NEAT1* expression, as chronic exposure to antipsychotic mediations may have affected gene expression even after cessation of treatment. Finally, gene expression studies were carried out on homogenates of brain tissue and cannot confidently indicate the specific cell type(s) in which gene expression was altered. Nevertheless, unsupervised hierarchical clustering indicated that *NEAT1* exhibits the same transcriptional profile as the large group of myelin-specific genes in the brain regions characterized by OLG/myelin gene expression deficit in the microarray studies of multiple brain regions from individuals with SZ.^22^

## METHODS

### Ethics statement and brain specimens

Postmortem brains, donated by the next of kin of deceased subjects participating in studies of SZ, were received by the Mount Sinai NIH Neurobiobank—ISMMS, Icahn School of Medicine at Mount Sinai. All assessments were approved by governing ISMMS review board. The specimen handling, neuropathology and diagnostic systems used for classifying human brains have been described extensively.^56,57^ The demographic characteristics of the SZ and control cohorts have been described previously^31,58^ and are shown in Table S1. All SZ subjects had been chronically hospitalized at Pilgrim Psychiatric Center (NY), or associated nursing homes for many years. All patients had thorough and structured neuropathologic characterization to rule out discernable neuropathologies such as Alzheimer's disease (AD), multi-infarct dementia, etc.^59^ None of the cases or controls had any history of licit or illicit drug abuse (tobacco use excepted). All subjects died of natural causes.

### Microarray analysis

The group composition, demographic characteristics and the procedures for RNA isolation and the microarrays using Affymetrix (Santa Clara, CA) HG-U133AB GeneChip® set were as described previously.^22,29,58^ Statistical comparisons were made using GeneSpring GX12 (Agilent Technologies, Santa Clara, CA). Significantly different probe-sets as defined by a Benjamini–Hochberg^60^ adjusted moderated *t*-test^61^ *P*-values < 0.05 were used for subsequent analyses.

### Mouse models used in the study

Brains of 2–4-month-old (mean age = 2.9 m.o.) Neat1 knockout mouse (Neat1^-/-^; RIKEN accession # CDB0773K) with corresponding background wild type were provided by Dr Nakagawa (RIKEN, Japan). Briefly, Neat1^-/-^ was generated by inserting lacZ and polyadenylation signals immediately downstream of the *Neat1* transcriptional start site.^8^ Brains of 2–3-month-old *quaking*- *qk*^*e5*^ homozygous mice with corresponding background wild type were gifts of Dr Monica J. Justice (Baylor College of Medicine, Houston, TX; now at SickKids, Toronto, Ontario, Canada). The *qk*^*e5*^ mutation was induced on 101/R1 DNA after treatment with *N*-ethyl-*N*-nitrosourea as described previously.^52^ All animal procedures adhered to the National Institutes of Health Guide for the Care and Use of Laboratory Animals and were approved by the Institutional Animal Care and Use Committee of the Icahn School of Medicine at Mount Sinai and JJ Peters VA Medical Center.

### RNA isolation, library construction, RNA-seq, and data analysis, WGCNA

Brain dissections of adult mice included the frontal cortex gray matter and the white matter (see Supplementary Materials). Total RNA was isolated using Maxwell 16 LEV simplyRNA Tissue kit (Promega, Madison, WI). RNA libraries were prepared from frontal cortex of Neat1^-/-^ mice and controls by depleting rRNA. RNA-seq quality control, alignment, and gene expression quantifications were performed as described.^62^ Mapping and quantification, normalized gene expression, covariates exploration and construction of WGCNA described in Supplementary Materials.

### Gene set enrichment analysis

GSEA was performed with cameraPR in the limma package.^63^ In order to avoid using arbitrary cutoffs to identify DEGs, gene set enrichments were evaluated by applying cameraPR to the differential expression t-statistics. Mouse genes were converted to human orthologs. Pathways and curated gene sets were derived from the molecular signatures database (MSigDB, v6.2 updated July 2018 Broad Institute), a collection of annotated gene sets.^64,65^ A *p*-value ≤ 0.05 was considered as statistically significant.

#### Real-time PCR

Procedures for complementary DNA preparation for qPCR have been described previously.^2,21^ The mRNA levels of myelin genes were measured by qPCR using TaqMan® gene expression assays (Table S11, ThermoFisher). For relative quantification of mRNA expression, geometric means were calculated using the standard curve method. Housekeeping genes (*GUSB*, *PPIA*, and *RPLP0* for human; *GAPDH* and *GUSB* for mouse) were used as the endogenous references. The optimal set of housekeeping genes was established in preliminary experiments by using geNorm algorithm (Biogazelle).

#### In situ hybridization

Tissue pretreatment/protease digestion, probes (human and mouse *NEAT1*-T6 and *SOX10*-T1) hybridization and amplification procedures were performed according to the manufacturer’s protocol (2-plex QuantiGene ViewRNA Assay, Affymetrix, CA).

#### Flow cytometry

Brain tissue disassociation, nuclei purification with sucrose gradient, and antibody staining were carried out according to previously published protocol.^54^ Primary antibodies: *Olig2-* AlexaFluor 488 (1:50 v/v, EMD Millipore MABN50A4, MO) and *NeuN*-AlexaFluor 647 (1:1000 v/v, Abcam ab190565, MA) were used. Flow cytometry was performed on a BD Melody (BD Biosciences, CA). The data were analyzed by the FCS Express 6 software (De Novo Software, CA). Doublets discrimination analysis was done based on signal processing (height vs. width). *Olig2*^+^ and *NeuN*^+^ cells were determined based on the AF488 and AF674 fluorescence, correspondently.

### Chromatin isolation by *NEAT1*-RNA purification, ChIRP

Nuclei purification from frozen postmortem human brain gray matter (BA4) were performed as described before.^54^ NEAT1-CHIRP assay was performed according to manufacturer protocol (NEAT1-EZ Magna ChIRP, EMD Millipore, MO). The *NEAT1* probes were divided into two pools: even and odd. RNA libraries of even-, odd-*NEAT1* pools, and negative control (LacZ) were constructed using SMARTer® Stranded Total RNA v2 - Pico (Takara Bio USA, CA). ChIRP-seq was performed on Illumina HiSeq 2500 system (GeneWiz, NJ).

#### Immunocytochemistry

Mouse brains were cut in 12 µm serial sections in the coronal plane. Tissue sections were postfixed and incubated with the primary antibodies overnight at 4 °C. Primary antibody against *Qki*-5 isoform (1:1000 v/v, gift of Dr Karen Artzt, Univ. of Texas at Austin and Dr Monica Justice, Baylor College of Medicine) with secondary anti-rabbit AF488 conjugated antibodies (1:1000 v/v, ThermoFisher A21206, CA) were used. DAPI (4′,6-diamidino-2-phenylindole)-counterstained sections were photographed using a Carl Zeiss AxioImager Z1 microscope and AxioVision Digital Image Processing System version 4.8.2.

### Statistics

Multiple statistical procedures were employed for different aspects of the study. Max *t*-scores, Pearson’s correlation coefficients, and corresponding *p*-values (*t*-test) for each individual transcript were calculated by the contrast analysis and described in details previously.^21^ Linear associations of gene expression with gender, age, pH, and PMI were assessed in exploratory analysis to evaluate their use as covariates. Effects of diagnosis on the dependent variables were examined by ANOVA. Student’s *t*-test was used to compare relative mRNA expression in qPCR experiments and analysis of immunostained tissue sections. All *p*-values refer to two-tailed probabilities. All procedures were performed using SPSS (IBM ver.22). A positional gene enrichment analysis of chromosomal regions^26^ was performed using a Web tool implementing this method applied to the human genome (http://www.esat.kuleuven.be/~bioiuser/pge).

### Reporting summary

Further information on research design is available in the Nature Research Reporting Summary.

## Supplementary information

Supplemental materials

Reporting summary

## Data Availability

All relevant data will be made available upon request. Raw microarray HG-U133AB GeneChip® set, RNA-seq Neat1 knockout dataset and RNA-seq Neat1 ChIRP set are available through a publically accessible website (https://harouv01.u.hpc.mssm.edu/).

## **Supplementary information**

**Supplementary information** accompanies the paper on the *npj Schizophrenia* website (10.1038/s41537-019-0071-2).
