# Supplementary material for: The expression of long noncoding RNA NEAT1 is reduced in schizophrenia and modulates oligodendrocytes transcription
Source: NPJ Schizophr. 2019 Jan 29;5:3. doi: 10.1038/s41537-019-0071-2 (PMC6386752; doi:10.1038/s41537-019-0071-2)
Supplement: Supplementary file 1 — Supplemental materials [file 41537_2019_71_MOESM1_ESM.pdf]

Table S1. Demographic and tissue preservation characteristics of the human microarray dataset [1] and independent validation cohort (qPCR).

	Microarray dataset		Hippocampus qPCR	
**Groups/arrays**	**Normal controls**	**Schizophrenia**	**Normal controls**	**Schizophrenia**
Subjects #	22	28	21	24
Total samples/arrays	233	273		
Sex (M/F)	8/14	19/9	8/13	13/11
Age (years)	81.6±11.6	74.5±11.2	79.7±2.4	75.8±2.1
Brain pH	6.5±0.2	6.4±0.2	6.5±0.1	6.4±0.1
PMI (h)	7.1±4.6	12.9±6.3	8.1±1.5	13.1±1.4

Table S2. Gene annotation for NEAT1 (chr11:65190269-65194003 (hg38)) for Affymetrix HG-U133AB microarray (https://genecards.weizmann.ac.il/cgi-bin/geneannot).

U133AB Array	Probe set	Sensitivity (1-highest)	Specificity (1-highest)	Genome locus- Chr11 (DNA strand)	NEAT1 variant
133B	224565_at	1	1	(+) 65191943- 65194000	short + long
133B	225239_at	2	0.778	(+) 65206882- 65208335	long

Table S3. Regional changes in mRNA levels of total NEAT1 (tNEAT1) and long NEAT1_22.7K isoform in cortical Brodmann areas and HIPP, and subcortical regions in individuals with SZ (Figure 1).

**Area**	$P^{\dagger}_{tNEAT1}$	$P_{NEAT1\_22.7K}$	$N_{Cnt/SZ}$
BA10	**0.004**	0.448	16/21
BA17	**0.024**	0.999	14/14
BA20	**0.048**	0.494	16/16
BA21	**0.004**	0.128	16/18
BA22	**0.019**	0.101	14/21
BA23/31	**0.012**	0.129	14/15
BA24/32	**0.043**	0.252	19/16
BA36/28	**0.001**	0.232	15/15
BA38	**0.0004**	0.248	14/14
BA4	**0.003**	0.219	6/16
BA44	**0.041**	0.261	13/16
BA46/9	**0.013**	0.234	17/27
BA7	**9.34E-05**	**0.014**	13/15
BA8	**0.019**	0.301	14/14
CD	**0.004**	0.529	11/15
HIPP	**0.013**	0.426	13/13
PT	**0.007**	0.300	9/11

† *p*-Values are from moderated t-test with Benjamini and Hochberg multiple testing corrections. Abbreviations: HIPP - hippocampus, CD -caudate and PT – putamen.

Table S4. Summary of regional changes of mRNA levels of NEAT1 total and long NEAT1_22,7K isoform in individuals with SZ (Fig. 1).

	NEAT1 total		NEAT1-22.7K		
Brain areas	*t* †	*p*	*t*	*p*	N= Cnt/SZ
**All cortex (17)**	10.3	**1.70E-16**	4.4	**4.02E-06**	233/273
Frontal	5.8	**3.58E-08**	2.2	**0.026**	65/90
Cingulate	3.3	**0.002**	1.9	0.063	33/31
Temporal	6.6	**7.24E-10**	2.9	**0.005**	75/84
Parietal	4.6	**9.34E-05**	2.6	**0.014**	13/15
Occipital	2.4	**0.024**	0.002	0.99	14/14
HIPP	2.7	**0.013**	0.8	0.43	13/13
Subcortical	4.4	**6.53E-05**	0.9	0.33	20/26

† t-scores were calculated by the contrast analysis (see Methods). *p*-Values are from moderated t-test with Benjamini and Hochberg multiple testing corrections. Bold text indicates statistically significant p values.

Table S5. Genes affected in murine Neat1 locus (Chr.19) in frontal cortex of Neat1 KO mice.

Symbol	*t*	*p-val*†	*Chr.*	*Location* *(negative strand)*
Cd6	-20.3	9.79E-11	19	10789470-10829832 (-)
Malat1*	-14.2	6.38E-09	19	5795690-5797754 (-)
**Neat1***	-13.7	9.02E-09	19	5830411-5831249 (-)
Map4k2	-9.2	7.43E-07	19	6341249-6353527
Bbs1	-7.7	5.01E-06	19	4886879-4889382 (-)
Pygm	-6.4	3.19E-05	19	6384415-6398459
Fkbp2	-5.6	0.0001	19	6977736-6979503 (-)
Brms1	-5.5	0.0001	19	5041371-5049912
Prpf19	-5.4	0.0002	19	10895230-10905530
Mrpl11	-4.6	0.0006	19	4962326-4964948
Ehd1*	-4.5	0.0007	19	6276874-6300096
Fads1	-4.4	0.0008	19	10182902-10196874
Cnih2	-4.4	0.0008	19	5092867-5098418 (-)
Plcb3	-4.3	0.0010	19	6953713-6969794 (-)
Wdr74	-4.3	0.0010	19	8735813-8740623
Prdx5	-4.1	0.0014	19	6906702-6910106 (-)
Pla2g16	-3.9	0.0019	19	7557519-7585785
Mus81*	5.9	6.92E-05	19	5482841-5488337 (-)
Slc3a2	11.4	7.45E-08	19	8707570-8713821 (-)
Rtn3	14.1	7.08E-09	19	7425900-7483226 (-)
Frmd8*	25.2	7.28E-12	19	5851581-5853036 (-)

* Homologous genes affected in human NEAT1 locus in SZ. Genes ranked by t-scores.
† p-Values are from moderated t-test adjusted using false discovery rate (FDR) estimation.

Table S6. Significant (p<0.001) DEGs located in human NEAT1 locus (Chr.11q13.1) between individuals with SZ and controls. Data derived from 17 brain regions microarray dataset [1,2] and NEAT1/ChIRP-seq.

Symbol	*t-score*†	*Chr.*	*Location* *(negative strand)*	*NEAT1 ChIRP-seq (counts)*
**NEAT1***	-10.3	11	65422798..65445540	580955
FRMD8*	-6.5	11	65386570..65413525	63
SYVN1	-5.2	11	65127279..65135178 (-)	160
EHD1*	-5	11	64852727..64879713 (-)	207
DPF2	-4.7	11	65333754..65352980	157
SF1	-4.5	11	64764604..64779043 (-)	968
MALAT1*	-3.4	11	65497679..65504494	98684
LTBP3	-2.9	11	65538559..65558388 (-)	798
TM7SF2	-2.7	11	65111854..65116235	122
KAT5	-2.7	11	65711996..65719606	69
RELA	-2.4	11	65653596..65662972 (-)	94
EIF1AD	-2.2	11	65996545..66002217 (-)	71
MUS81*	2.7	11	65860244..65866443	55
SNX15	2.8	11	65027408..65040572	-
FAM89B	3.1	11	65572349..65574198	-
CAPN1	3.3	11	65181215..65212006	102
CCDC85B	3.7	11	65890404..65891635	-
KLC2	3.9	11	66257294..66267861	172
NRXN2	4.9	11	64606174..64723188 (-)	438

* Homologous genes affected in mouse NEAT1 locus in frontal cortex Neat1 KO mice.

† t-scores were calculated by the contrast analysis (see Methods).

Table S7. Key pathways, pathway groups and the most relevant networks enriched in frontal cortex of Neat1$^{-/-}$ mice.

**Key Pathways**	***P***	**FDR**
Regulation of lipid metabolism via LXR, NF-Y and SREBP	2.5E-06	0.0009
C5a signaling	7.7E-06	0.001
Role of cell-cell and ECM-cell interactions in OLG differentiation/ myelination	5.8E-05	0.003
VEGF-family signaling	3.8E-05	0.004
TGF, WNT and cytoskeletal remodeling	6.3E-05	0.005

**Pathway groups**	**Input Data *P***	**Key Hubs *P***	**Union *P***
Cell differentiation	0.01	1.2E-08	1.5E-09
Angiogenesis	0.01	1.7E-06	9.7E-08
Apoptosis	0.03	1.8E-05	3.2E-06
Tissue remodeling and wound repair	0.04	0.0007	0.0001
TGF, WNT and cytoskeletal remodeling	0.02	0.002	0.0001
Epigenetic and transcriptional regulation of OLG precursor cell differentiation and myelination	0.01	0.03	0.001

**Network name**	**Processes**	**Pathways**	***P***	**gScore**
Dsh, Irs2, Wnt, Apc protein, Id2	Canonical Wnt signaling pathway (43.8%), regulation of Wnt signaling pathway (54.2%), regulation of cell proliferation (81.2%)	260	3E-12	338.78
Akt, PI3K reg. (p85), Adenylate cyclase, p90Rsk	Positive regulation of metabolic process (89.1%), cell differentiation (84.8%)	72	1E-14	105.69
Col IV, Akt, Wnt, Apc protein, Serpine2	Blood vessel development (56.8%), cell migration (63.6%), cell motility (63.6%)	46	6E-07	67.95

Table S8. RNA processing category (*greenyellow* module) differentially expressed genes in frontal cortex of Neat1 null mice identified by IPA and their expression in human NEAT1/ChIRP-seq.

Symbol	*Gene Name*	*t-scores*	*p-val*	*hNEAT1 ChIRP-seq (counts)*
MALAT1	metastasis associated lung adenocarcinoma transcript 1	-14.18	6.38E-09	98684
PRPF19	pre-mRNA processing factor 19	-5.37	1.6E-04	262
INTS10	integrator complex subunit 10	-4.10	0.001	286
RBMS2	RNA binding motif single stranded interacting protein 2	-2.73	0.018	190
HEATR1	HEAT repeat containing 1	-2.69	0.020	282
NPM3	nucleophosmin/nucleoplasmin 3	-2.65	0.021	-
SNW1	SNW domain containing 1	-2.54	0.026	135
USP39	ubiquitin specific peptidase 39	-2.52	0.026	124
POLR2A	RNA polymerase II subunit A	-2.51	0.027	533
UTP20	UTP20, small subunit processome component	-2.48	0.029	285
SF3B3	splicing factor 3b subunit 3	-2.36	0.036	377
YTHDC1	YTH domain containing 1	-2.29	0.041	-
HBB	hemoglobin subunit beta	-2.29	0.041	442
WDR12	WD repeat domain 12	-2.25	0.044	148
ERN1	endoplasmic reticulum to nucleus signaling 1	-2.13	0.054	54
IMP3	IMP3, U3 small nucleolar ribonucleoprotein	-2.13	0.054	-
TSEN54	tRNA splicing endonuclease subunit 54	-2.04	0.064	-
PAPOLA	poly(A) polymerase alpha	-2.03	0.065	318
DIS3	DIS3 homolog, exosome endoribonuclease	2.02	0.066	390
PCBP4	poly(rC) binding protein 4	2.13	0.054	167
LSM5	LSM5 homolog, U6 small nuclear RNA/mRNA degradation associated	2.20	0.048	154
TBP	TATA-box binding protein	2.22	0.046	1040
LAS1L	LAS1 like, ribosome biogenesis factor	2.22	0.046	135
HNRNPA0	heterogeneous nuclear ribonucleoprotein A0	2.32	0.038	175
RPS15	ribosomal protein S15	2.32	0.038	88
RBFOX3	RNA binding protein, fox-1 homolog (C. elegans) 3	2.35	0.037	547
SART3	squamous cell carcinoma antigen recognized by T-cells 3	2.40	0.034	109
SRSF4	serine and arginine rich splicing factor 4	2.52	0.027	204
RPS19	ribosomal protein S19	2.53	0.026	113
RBFOX1	RNA binding protein, fox-1 homolog 1	2.58	0.024	510
DYRK1A	dual specificity tyrosine phosphorylation regulated kinase 1A	2.62	0.022	329
HNRNPU	heterogeneous nuclear ribonucleoprotein U	2.67	0.020	1339
PLCB1	phospholipase C beta 1	2.83	0.015	967
RPL5	ribosomal protein L5	2.89	0.013	326
IWS1	IWS1, SUPT6H interacting protein	2.90	0.013	299
RNASEL	ribonuclease L	2.97	0.012	102
RPL7	ribosomal protein L7	3.00	0.011	321

RPS16	ribosomal protein S16	3.08	0.009	99
XRN1	5'-3' exoribonuclease 1	3.30	0.006	286
RBM3	RNA binding motif (RNP1, RRM) protein 3	3.36	0.006	83
CPEB1	cytoplasmic polyadenylation element binding protein 1	3.63	0.003	155
RPS7	ribosomal protein S7	3.64	0.003	139
RPL14	ribosomal protein L14	3.91	0.002	110
JUN	Jun proto-oncogene, AP-1 transcription factor subunit	5.19	2.17E-04	70

Table S9. The list of curated gene sets identified by GSEA in frontal cortex of Neat1 null mice.

Curated / GO/ Hallmark Gene sets	N Genes	Dir	p-val	FDR
c2.SCHMIDT_POR_TARGETS_IN_LIMB_BUD_UP	22	Up	2.24E-12	4.1E-08
c2.HORTON_SREBF_TARGETS	23	Up	7.26E-10	6.1E-06
c2.REACTOME_PEPTIDE_CHAIN_ELONGATION	53	Up	1.32E-09	6.1E-06
Peptide_chain_elongation	53	Up	1.32E-09	6.1E-06
c2.REACTOME_CHOLESTEROL_BIOSYNTHESIS	19	Up	2.39E-09	8.9E-06
Cholesterol_biosynthesis	20	Up	3.57E-09	9.5E-06
viral transcription	54	Up	3.57E-09	9.5E-06
Eukaryotic_Translation_Termination	54	Up	4.50E-09	1.1E-05
Eukaryotic_Translation_Elongation	56	Up	7.56E-09	1.5E-05
Viral_mRNA_Translation	55	Up	1.02E-08	1.9E-05
c2.FERRANDO_TAL1_NEIGHBORS	7	Down	1.98E-08	3.3E-05
c2.KEGG_RIBOSOME	55	Up	2.13E-08	3.3E-05
Formation_of_a_pool_of_free_40S_subunits	64	Up	4.79E-08	6.6E-05
cytosolic small ribosomal subunit	22	Up	4.98E-08	6.6E-05
cytosolic ribosome	59	Up	5.36E-08	6.7E-05
translational termination	58	Up	8.24E-08	9.6E-05
viral genome expression	63	Up	1.10E-07	0.0001
Nonsense_Mediated_Decay_Independent_of_the_Exon_Junction_Complex	59	Up	1.86E-07	0.0002
c2.BILANGES_SERUM_AND_RAPAMYCIN_SENSITIVE_GENES	48	Up	9.02E-07	0.001
cholesterol biosynthetic process	29	Up	9.27E-07	0.001
translational elongation	71	Up	1.21E-06	0.001
GTP_hydrolysis_and_joining_of_the_60S_ribosomal_subunit	74	Up	1.29E-06	0.001
cytosolic large ribosomal subunit	34	Up	1.49E-06	0.001
c2.REACTOME_3_UTR_MEDIATED_TRANSLATIONAL_REGULATION	73	Up	2.00E-06	0.001
3'_-UTR-mediated_translational_regulation	73	Up	2.00E-06	0.001
L13a-mediated_translational_silencing_of_Ceruloplasmin_expression	73	Up	2.00E-06	0.001
cellular protein complex disassembly	72	Up	4.28E-06	0.003
c5.GO_CYTOSOLIC_RIBOSOME	72	Up	6.35E-06	0.004
c2.WANG_TNF_TARGETS	8	Down	6.63E-06	0.004
c5.GO_STEROL_BIOSYNTHETIC_PROCESS	32	Up	7.94E-06	0.005
c5.GO_INFLAMMATORY_RESPONSE_TO_ANTIGENIC_STIMULUS	9	Down	8.63E-06	0.005
sterol biosynthetic process	32	Up	8.71E-06	0.005
c5.GO_CYTOSOLIC_LARGE_RIBOSOMAL_SUBUNIT	38	Up	9.82E-06	0.006
Activation_of_Gene_Expression_by_SREBP_(SREBF)	39	Up	1.35E-05	0.007
protein complex disassembly	74	Up	1.61E-05	0.009
c2.REACTOME_INFLUENZA_VIRAL_RNA_TRANSCRIPTION_AND_REPLICATION	69	Up	1.77E-05	0.009
Influenza_Viral_RNA_Transcription_and_Replication	70	Up	2.15E-05	0.011
c2.REACTOME_formation_of_the_ternary_complex_and_subsequently_the_43s_complex	34	Up	2.32E-05	0.011
Formation_of_the_ternary_complex_and_subsequently_the_43S_complex	34	Up	2.32E-05	0.011
Cap-dependent_Translation_Initiation	81	Up	2.51E-05	0.011

Eukaryotic_Translation_Initiation	81	Up	2.51E-05	0.011
c5.GO_CYTOSOLIC_SMALL_RIBOSOMAL_SUBUNIT	27	Up	2.67E-05	0.012
c5.GO_ESTABLISHMENT_OF_PROTEIN_LOCALIZATION_TO_ENDOPLASMIC_RETICULUM	72	Up	3.58E-05	0.016
SRP-dependent cotranslational protein targeting to membrane	74	Up	3.69E-05	0.016
c2.REACTOME_NONSENSE_MEDIATED_DECAY_ENHANCED_BY_THE_EXON_JUNCTION_COMPLEX	75	Up	3.83E-05	0.016
Nonsense-Mediated_Decay	76	Up	4.39E-05	0.017
Nonsense_Mediated_Decay_Enhanced_by_the_Exon_Junction_Complex	76	Up	4.39E-05	0.017
c2.GESERICK_TERT_TARGETS_DN	20	Down	9.14E-05	0.036
viral infectious cycle	95	Up	9.71E-05	0.037
cotranslational protein targeting to membrane	76	Up	0.0001	0.038
c2.LEIN_OLIGODENDROCYTE_MARKERS	70	Down	0.000127	0.043
c2.REACTOME_SRP_DEPENDENT_COTRANSLATIONAL_PROTEIN_TARGETING_TO_MEMBRANE	76	Up	0.000135	0.049
SRP-dependent_cotranslational_protein_targeting_to_membrane	76	Up	0.000135	0.049
Ribosomal_scanning_and_start_codon_recognition	41	Up	0.000147	0.052

Table S10. Oligodendrocyte lineage specific genes detected by human NEAT1-ChIRP-seq (http://web.stanford.edu/group/barres_lab/tmp_rnaseq.html).

Entrez Gene Name	Gene Symbol	hNEAT1 ChIRP-seq (counts)
myelin basic protein	MBP	3701
proteolipid protein 1	PLP1	3258
protocadherin 9	PCDH9	2581
neurofascin	NFASC	2534
ankyrin 3	ANK3	2514
ATP binding cassette subfamily A member 2	ABCA2	2504
KIAA0930	KIAA0930	2284
kinesin family member 5A	KIF5A	2016
QKI, KH domain containing RNA binding	QKI	1930
dedicator of cytokinesis 5	DOCK5	1700
microtubule associated monooxygenase, calponin and LIM domain containing 3	MICAL3	1650
solute carrier family 4 member 10	SLC4A10	1455
myelin-associated oligodendrocyte basic protein	MOBP	1255
RAP1 GTPase activating protein	RAP1GAP	1198
tubulin tyrosine ligase like 7	TTLL7	1141
ubiquitin specific peptidase 54	USP54	1097
contactin 2	CNTN2	1079
dipeptidyl peptidase like 6	DPP6	988
SRC kinase signaling inhibitor 1	SRCIN1	988
growth arrest specific 7	GAS7	981
plakophilin 4	PKP4	962
pleckstrin homology, MyTH4 and FERM domain containing H1	PLEKHH1	961
crystallin alpha B	CRYAB	938
myelin regulatory factor	MYRF	927
pleckstrin homology like domain family B member 1	PHLDB1	924
ankyrin repeat and sterile alpha motif domain containing 1B	ANKS1B	899
tubulin polymerization promoting protein	TPPP	862
stearoyl-CoA desaturase	SCD	847
contactin 1	CNTN1	842
transferrin	TF	804
nicotinamide nucleotide adenylyltransferase 2	NMNAT2	778
DS cell adhesion molecule	DSCAM	770
versican	VCAN	768
peroxisomal biogenesis factor 5 like	PEX5L	755
semaphorin 4D	SEMA4D	753
prostaglandin D2 synthase	PTGDS	743
ArfGAP with GTPase domain, ankyrin repeat and PH domain 1	AGAP1	733
N-myc downstream regulated 1	NDRG1	723

ectonucleotide pyrophosphatase/phosphodiesterase 2	ENPP2	722
Ras protein specific guanine nucleotide releasing factor 1	RASGRF1	717
synaptotagmin like 2	SYTL2	717
neural EGFL like 2	NELL2	713
Rap guanine nucleotide exchange factor 4	RAPGEF4	708
cytoplasmic FMR1 interacting protein 2	CYFIP2	707
dipeptidyl peptidase like 10	DPP10	702
SECIS binding protein 2 like	SECISBP2L	690
solute carrier family 5 member 11	SLC5A11	687
cellular repressor of E1A stimulated genes 2	CREG2	642
ST18, C2H2C-type zinc finger	ST18	632
ryanodine receptor 3	RYR3	607
synaptotagmin 11	SYT11	598
transforming acidic coiled-coil containing protein 2	TACC2	595
fibrillin 1	FBN1	586
trafficking kinesin protein 2	TRAK2	568
sodium voltage-gated channel alpha subunit 3	SCN3A	566
chromosome 1 open reading frame 21	C1orf21	560
pleckstrin homology domain containing A1	PLEKHA1	560
extracellular leucine rich repeat and fibronectin type III domain containing 2	ELFN2	559
dedicator of cytokinesis 10	DOCK10	552
amyloid beta precursor protein binding family B member 1	APBB1	541
tenascin R	TNR	538
acetoacetyl-CoA synthetase	AACS	531
glutamate ionotropic receptor AMPA type subunit 3	GRIA3	528
cyclin dependent kinase 18	CDK18	526
kinase non-catalytic C-lobe domain containing 1	KNDC1	525
CUB and Sushi multiple domains 3	CSMD3	521
adenosylmethionine decarboxylase 1	AMD1	520
immunoglobulin superfamily member 8	IGSF8	518
synaptotagmin 16	SYT16	513
Janus kinase and microtubule interacting protein 3	JAKMIP3	508
2',3'-cyclic nucleotide 3' phosphodiesterase	CNP	506
breast carcinoma amplified sequence 1	BCAS1	502
peptidyl arginine deiminase 2	PADI2	492
TBC1 domain family member 9B	TBC1D9B	485
fasciculation and elongation protein zeta 1	FEZ1	484
sodium voltage-gated channel alpha subunit 1	SCN1A	482
G protein-coupled receptor class C group 5 member B	GPRC5B	480
protocadherin related 15	PCDH15	479
pleckstrin homology domain containing B1	PLEKHB1	475
proprotein convertase subtilisin/kexin type 6	PCSK6	473

phosphatase and actin regulator 1	PHACTR1	469
potassium voltage-gated channel subfamily H member 8	KCNH8	467
SH3 domain containing 19	SH3D19	457
glutamate ionotropic receptor AMPA type subunit 4	GRIA4	439
neurexin 2	NRXN2	438
solute carrier family 24 member 4	SLC24A4	438
SCO-spondin	SSPO	430
cordon-bleu WH2 repeat protein	COBL	427
amyloid beta precursor like protein 1	APLP1	423
kinesin family member 13B	KIF13B	419
inositol 1,4,5-trisphosphate receptor type 2	ITPR2	407
plexin B3	PLXNB3	404
synaptojanin 2	SYNJ2	403
transmembrane protein 132B	TMEM132B	399
phosphofurin acidic cluster sorting protein 2	PACS2	395
DnaJ heat shock protein family (Hsp40) member B2	DNAJB2	392
striatin	STRN	391
BCAR1, Cas family scaffolding protein	BCAR1	390
N-acetylglucosamine-1-phosphate transferase alpha and beta subunits	GNPTAB	385
malic enzyme 3	ME3	385
glutamic--pyruvic transaminase 2	GPT2	377
family with sequence similarity 131 member B	FAM131B	376
proprotein convertase subtilisin/kexin type 2	PCSK2	376
DIX domain containing 1	DIXDC1	375
discoidin domain receptor tyrosine kinase 1	DDR1	360
glutamate ionotropic receptor kainate type subunit 1	GRIK1	360
SLIT and NTRK like family member 4	SLITRK4	350
solute carrier family 44 member 1	SLC44A1	349
unc-5 netrin receptor C	UNC5C	348
anillin actin binding protein	ANLN	346
carnosine synthase 1	CARNS1	344
adenosine monophosphate deaminase 3	AMPD3	336
leucine rich repeat transmembrane neuronal 4	LRRTM4	335
MON2 homolog, regulator of endosome-to-Golgi trafficking	MON2	332
epsin 2	EPN2	331
shisa family member 7	SHISA7	330
mitogen-activated protein kinase 8 interacting protein 1	MAPK8IP1	326
PDZ and LIM domain 2	PDLIM2	322
transcription elongation factor A like 6	TCEAL6	322
glutamate ionotropic receptor kainate type subunit 4	GRIK4	321
family with sequence similarity 102 member A	FAM102A	320
fidgetin, microtubule severing factor	FIGN	318
zinc finger protein 365	ZNF365	318

calcium voltage-gated channel auxiliary subunit beta 4	CACNB4	314
MCF.2 cell line derived transforming sequence	MCF2	314
nudix hydrolase 16	NUDT16	312
exostosin like glycosyltransferase 1	EXTL1	301
disco interacting protein 2 homolog A	DIP2A	300
erb-b2 receptor tyrosine kinase 3	ERBB3	300
leucine rich repeat transmembrane neuronal 2	LRRTM2	298
myelin associated glycoprotein	MAG	296
S100 calcium binding protein B	S100B	285
integrin subunit beta 8	ITGB8	282
sorting nexin family member 30	SNX30	273
oxysterol binding protein like 1A	OSBPL1A	264
ermin	ERMN	257
ArfGAP with coiled-coil, ankyrin repeat and PH domains 3	ACAP3	255
SET binding factor 1	SBF1	251
myelin oligodendrocyte glycoprotein	MOG	250
dynein light chain LC8-type 2	DYNLL2	245
catenin alpha 3	CTNNA3	241
1-acylglycerol-3-phosphate O-acyltransferase 4	AGPAT4	239
seizure related 6 homolog like	SEZ6L	233
KN motif and ankyrin repeat domains 1	KANK1	226
oligodendrocyte transcription factor 1	OLIG1	221
protein phosphatase 1 regulatory subunit 16B	PPP1R16B	221
inositol-tetrakisphosphate 1-kinase	ITPK1	220
neurocalcin delta	NCALD	216
hedgehog interacting protein	HHIP	206
integrin subunit beta 4	ITGB4	205
oligodendrocytic myelin paranodal and inner loop protein	OPALIN	201
glutamate ionotropic receptor kainate type subunit 3	GRIK3	187
apoptosis associated tyrosine kinase	AATK	172
apolipoprotein D	APOD	170
claudin 11	CLDN11	167
cysteine and glycine rich protein 1	CSRP1	156
FYN proto-oncogene, Src family tyrosine kinase	FYN	154
pleckstrin homology and RhoGEF domain containing G3	PLEKHG3	152
KIAA1147	KIAA1147	149
F-box protein 32	FBXO32	141

Table S11. List of TaqMan assays used in this study.

Gene symbol	Gene Name (Human)	Assay ID (Hs_)	NCBI (NM_)	Target Exon	
NEAT1	nuclear-enriched abundant transcript 1	01008264_s1	AF080092	1	lncRNA
GUSB	glucuronidase, beta	99999908_m1	000181.3	11-12	Normalization standard
PPIA	peptidylprolyl isomerase A	04194521_s1	021130.3	5	Normalization standard
RPLP0	large ribosomal protein P0	99999902_m1	053275.3	1-1	Normalization standard
Gene symbol	Gene Name (Mouse)	Assay ID (Mm_)	NCBI (NM_)	Target Exon	Role in OLG/myelin
Cldn11	claudin 11	00500915_m1	008770	2-3	Tight junction protein, myelin sheath compaction
Cnp	cyclic nucleotide phosphodiesterase 1	01306640_m1	1146318.1	2-3	Myelin-associated enzyme, early OLG marker
Gapdh	glyceraldehyde-3-phosphate dehydrogenase	99999915_g1	001001303	2-3	Glycolysis
Gusb	glucuronidase, beta	00446956_m1	010368.1	4-5	Carbohydrates metabolism
Mag	myelin association glycoprotein	00487538_m1	10758.2	5-6	Myelin adhesion glycoprotein
Mbp	myelin basic protein	01266402_m1	010777	5	Major constituent of the myelin sheaths
Neat1	nuclear-enriched abundant transcript 1	03455878_s1	NR_003513.2	1	Unknown
Olig2	oligodendrocyte transcription factor 2	01210556_m1	016967	1-2	Transcription factor, OPC/OLG marker
Plp1	proteolipid protein (myelin) 1	00456892_m1	011123.2	2-3	Myelin trans-membrane protein, myelin compaction
Pspc1	paraspeckle protein 1	00481804_m1	025682.3	1	Unknown
Qki5/6	quaking 6 and 5b isoforms	01318927_m1	001159516.1	5	Nuclear RNA-binding protein
Qki7	quaking 7a isoform	01318925_m1	021881	6-7	Nuclear/Cytoplasmic RNA-binding protein
Sox10	SRY-box containing gene 10	01300162_m1	011437.1	3	Transcription factor, OPC/OLG marker

Table S12. Gene expression changes for OLG-specific genes in the frontal cortex of Neat1$^{-/-}$ mice as determined by RNA-seq and qPCR (Figure 4C) analyses.

Symbol	*Fold Change RNA-Seq*	*p-val*	*Fold Change qPCR*	*p-val*
Cldn11	-2.1	0.05*	-1.4	0.06
Cnp	-1.7	0.09	-1.5	0.02
Mag	-1.7	0.05	-1.7	0.002
Mbp	-1.5	0.29	-1.4	0.34
Olig2	-2.4	0.001	-2.3	0.01
Plp1	-1.7	0.13	-1.7	0.23
Qki	1.1	0.56	-	-
Qki5/6	-	-	2.7	0.01
Qki7	-	-	1.5	0.22
Sox10	-1.5	0.15	-1.4	0.03

* Significant t-test p-val are highlighted.

Figure S1. Gene expression levels of total *NEAT1* in hippocampus of individuals with schizophrenia (SZ, N=24) and those free of neuroleptic medications (range 4 weeks – 7 years; SZoff, N=5), and normal controls (NL, N=21). Relative expression values (qPCR) were normalized to geometric means of *GUSB, RPLP0* and *PPIA*. NEAT1 expression was significantly reduced in both SZ groups relative to controls (ANOVA, all p<0.05).

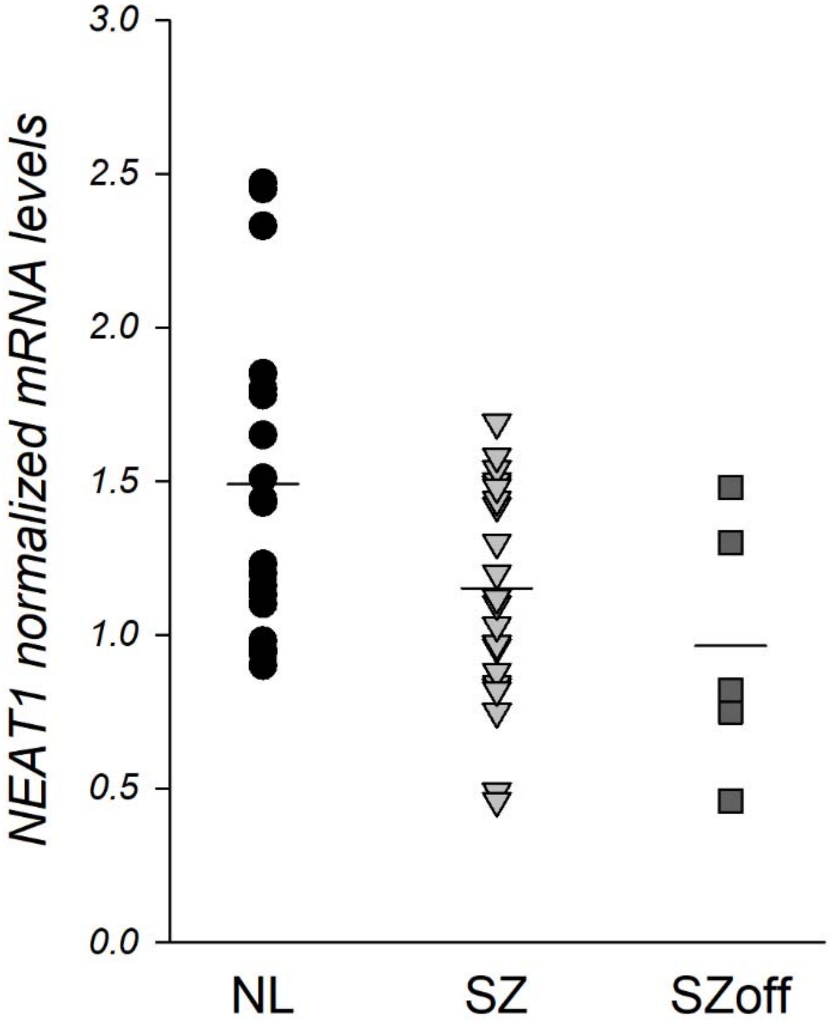

Figure S2. A positional gene enrichment analysis [3] of human homologs of Neat1$^{-/-}$ DEGs confirmed that the region centered around *NEAT1* on human chromosome 11 had the highest enrichment score (168.5; the probability of having the observed number of DEGs in the region was calculated by the hypergeometric distribution, adjusted p-val were calculated using the minimum p-val cumulative distribution function; p_{adj} = 1.83E-14).

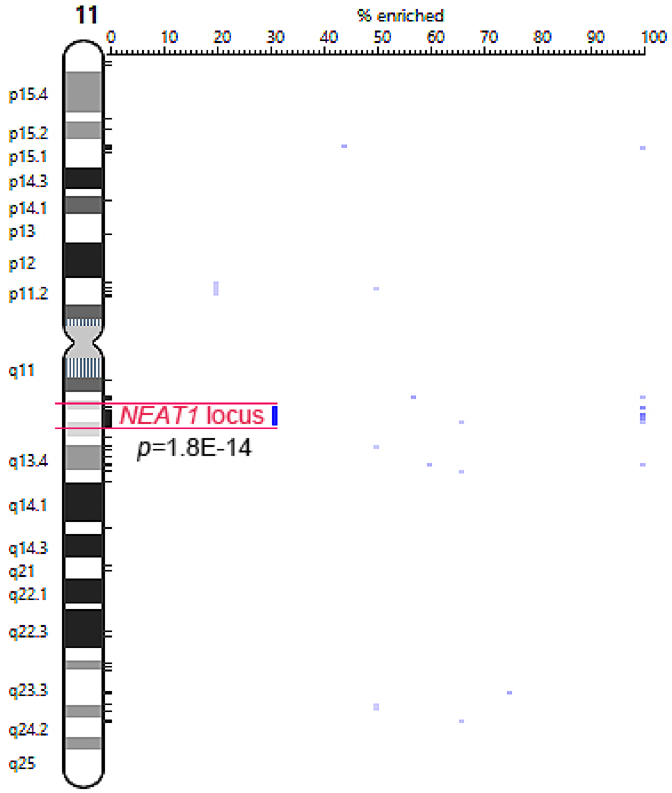

Figure S3. Normalized mRNA levels of Neat1 in the frontal cortex of Neat1$^{-/-}$ mice as determined by qPCR (n=5/group). Mean ± SEM is shown.

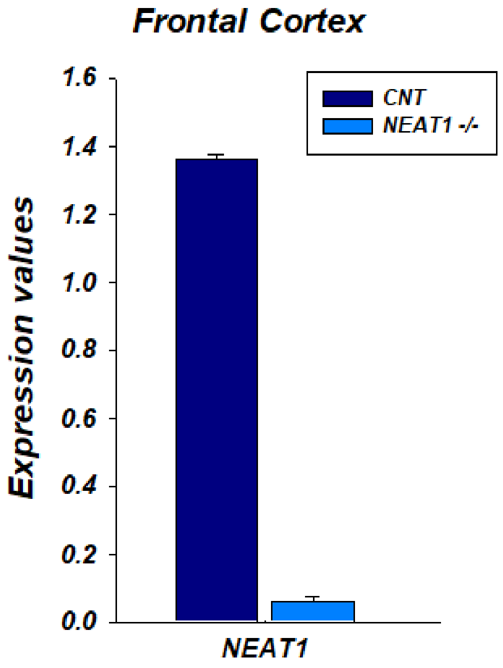

Figure S4. Gene co-expression networks in murine frontal cortex. Affected genes influencing processing of RNA (Table S8) identified by IPA. Colored labels indicate upregulation (red), or downregulation (green). Blue arrows and shapes indicate predicted inhibition; yellow-prediction influence insufficient. Values are t_s and t-test p values.

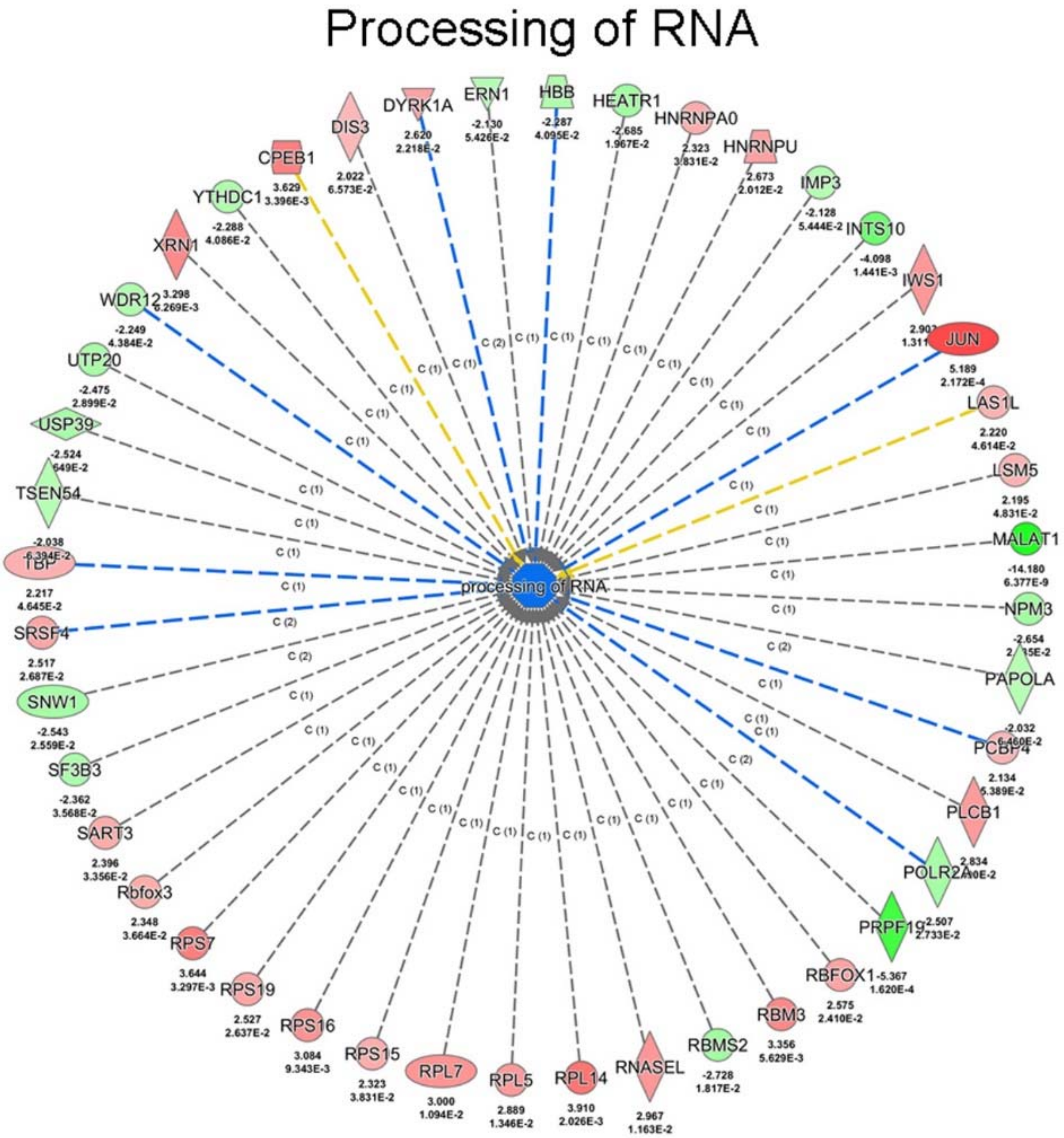

Figure S5. Affected genes influencing OLG differentiation (Figure 4B) identified by Ingenuity Pathway Analysis in Alzheimer's disease as a function of increased neurofibrillary tangles pathology (Braak staging)[4]. Colored labels indicate upregulation (red), or downregulation (green). Orange shape indicates predicted activation. Values are t-scores and p values shown in Table 1.

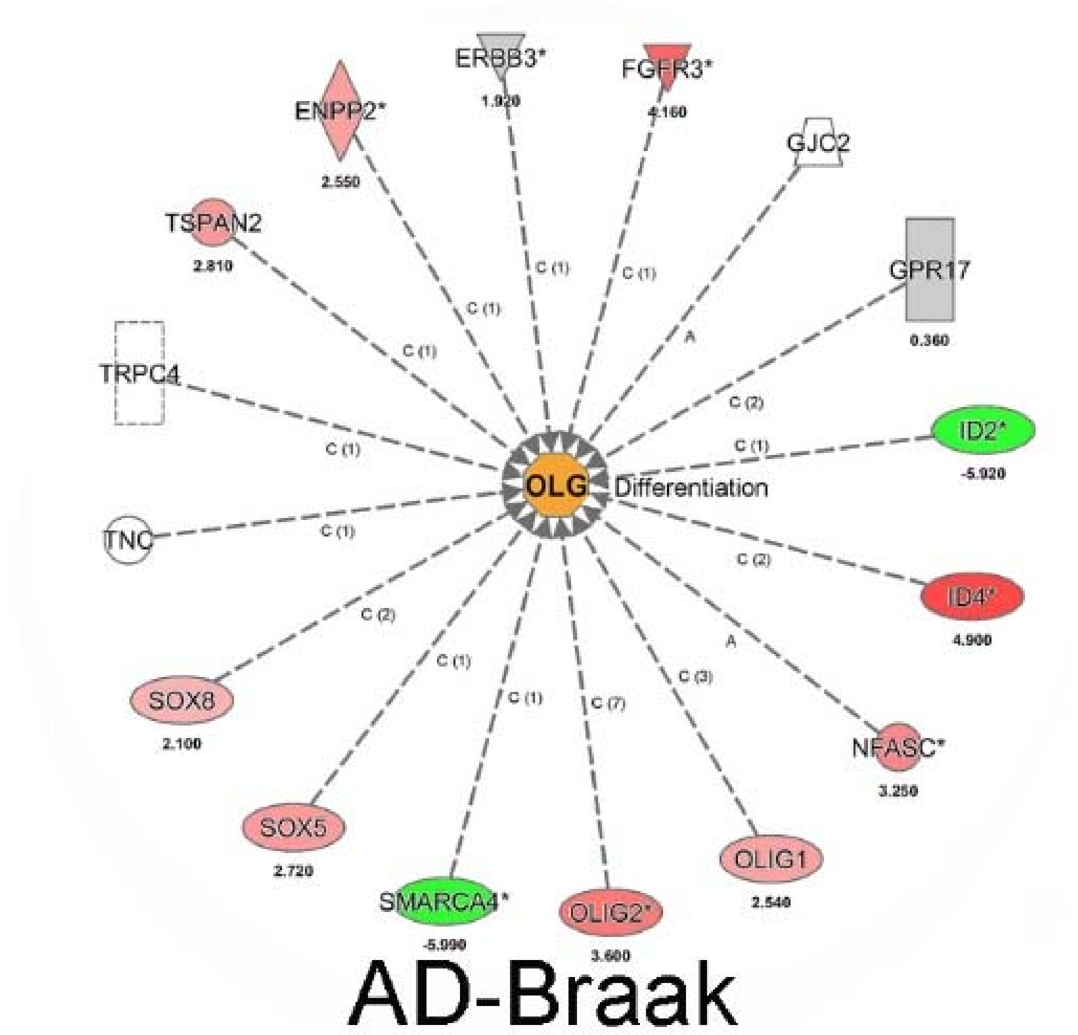

SUPPLEMENT METHODS.

Brain Specimens. Postmortem brains, donated by the next of kin of deceased subjects participating in studies of schizophrenia, were received over a period of 20 years by the Mount Sinai NIH Neurobiobank – ISMMS, Icahn School of Medicine at Mount Sinai. All assessments were approved by governing ISMMS review board and the next of kin of all tissue donors gave formal written consent for research use of the brain tissue. The specimen handling, neuropathology and diagnostic systems used for classifying human brains have been described extensively [5,6]. Briefly, brains were removed as soon after death as possible and were divided in half, sagittally. The right half was fixed in paraformaldehyde for neuropathological examination, diagnosis and anatomical studies. The left half was sectioned coronally into 0.8 cm blocks, snap-frozen and stored -80°C for further dissection. For this study, an approximately 1cc block of grey matter from the snap-frozen superior temporal gyrus was dissected at mid-level while still frozen and pulverized in a liquid nitrogen cooled mortar and pestle and divided into 50 mg aliquots.

RNA Isolation, library construction, RNA-seq and data analysis. The frontal cortex grey matter (from the frontal pole to the beginning of the parietal cortex (bregma -1.8 [7]); white matter - the corpus callosum underlying the dissected frontal cortical grey matter were used. The border of the frontal cortex and posterior cortical regions was identified as the coronal block where the middle cerebral artery bifurcates from the internal carotid artery [8] and the dissections were limited to the cortical region dorsal to the rhinal fissure. Total RNA was isolated using Maxwell 16 LEV simplyRNA Tissue kit (Promega). Coding and noncoding RNA library were prepared from frontal cortex of Neat1$^{-/-}$ mice and controls by depleting rRNA. RNA-seq quality control, alignment and gene expression quantification were performed as described[9].

Mapping and quantification: Reads were mapped to mm10 reference genome using TopHat (version 2.0.9) and Bowtie (version 2.1.0), with the default parameters. Known iGenomes Ensembl mm10 were quantified by HTSeq (version 0.6.0) in intersection-strict mode. A sample-by-gene read count matrix was generated for all samples by the Ensembl genes. Scaling normalization to remove composition biases in sequencing data was applied to log(CPM) (read **C**ounts **P**er **M**illion total reads) using the trimmed mean of M-values (TMM) method[10].

Covariates exploration: We selected covariates for inclusion to differential gene expression analysis by performing principal component analysis of the normalized read count matrix and examining which variables were significantly correlated with the high-variance components (explaining > 1% of the variance) of the data. This identified two covariates: Group (knock out and wild type) and age.

Normalized gene expression: We used the voomWithQualityWeights function from the limma package to model the normalized read counts. This function estimates weights by combining observational-level weights from voom with sample-specific weights estimated using the arrayWeights function. Briefly, in the first step voom estimates confidence weights for each normalized observed read count by residualizing on the covariates, fitting a mean-variance relationship function across all genes, using the fitted function to estimate the variance of a particular read count observation, and then setting the observation weight to be the inverse of the corresponding estimated variance.This is an important step to model the statistical sampling of gene expression level, since larger log(CPM) typically exhibit lower variance. In the second step, the estimated sample weights are used to obtain observational weights that take into account variations in sample quality. The variance model is then fitted a second time and a final set of modified weights is used in the linear modeling and differential expression analysis.

Differential analysis. For each transcript, we fit weighted least-squares linear regression models for the effect on gene expression of each variable on the right-hand side:

Gene expression ~ Group + covariates

Then, for each transcript, the group (knock out and wild type) coefficient was statistically tested for being non-vanishing, implying an estimated effect, above and beyond any other effect from the covariates (age) on gene expression using linear regression utilities in the limma package[11]. P-values were then adjusted for multiple hypotheses testing using false discovery rate (FDR) estimation, and the differentially expressed genes were determined as those with an estimated FDR ≤ 0.05. Principal component analysis (PCA) was carried out using the PCA module of GenePattern software. The analysis identifies patterns by finding principal components that account for variance in data points among all samples. Each principal component thus represents a combination of gene expression values, and each principal component is mutually uncorrelated and orthogonal. Two principal component scores (PC1 and PC2) resolved 99% of variance amongst all samples. A two-dimensional plot of PC1 and PC2 was generated using the PCAviewer module of GenePattern.

Weighted gene co-expression analysis. Weighted gene co-expression networks were constructed using the WGCNA package in R starting with the normalized and residualized (removing effect of age) expression data. The connectivity metric between a pair of genes *i* and *j*, or k_{ij}, is a transformed correlation between their expression profiles, with the matrix $A = (k_{ij})$ known as the unsigned adjacency matrix. k_{ij} is defined as $|r_{ij}|^{\beta}$, using the absolute value of r_{ij}, the Pearson correlation coefficient between the profiles of genes *i* and *j*, and β is the parameter of a power function. β is selected using the fitting index proposed by Zhang et al. [12], i.e., to maximize the scale-free topology model fitting index R^2 of the linear model that regresses $log(p(k))$ on $log(k)$, where *k* is connectivity and $p(k)$ is the frequency distribution of connectivity.

To explore the modular structures of the coexpression network, the adjacency matrix is further transformed into a topological overlap matrix [13]. Use of the topological overlap metric leads to more cohesive and biologically meaningful modules, since it not only represents the direct correlation between two genes but also incorporates their indirect interactions through other genes in the network [12,13]. Genes were organized into discrete modules of highly coregulated genes (either correlated or anti-correlated) using hybrid dynamic tree-cutting algorithm to dynamically cut clustering dendrogram branches into discrete subsets of gene modules [14]. The minimum module size was set to 30 genes and the minimum height for merging

modules was set at 0.3. Ordered from largest (the module containing the most genes) to smallest, each module is sequentially assigned a color name. The less well-connected genes are arbitrarily grouped in the gray module.

Chromatin isolation by *NEAT1*-RNA purification, ChIRP. Tissue disassociation from frozen postmortem human brain grey matter (BA4), nuclei purification with sucrose gradient and ultracentrifugation were performed as described before [15]. NEAT1-CHIRP assay was performed according to manufacturer protocol (NEAT1-EZ Magna ChIRP, EMD Millipore, MO). DNA sonication conditions for DNA sizes <500bp was optimized and carried out for 2 hour in water bath (4^0C) using Q800R sonicator (Qsonica, CT). The probe design for human *NEAT1* lncRNA was based on the following parameters: 1 probe /100 bp of RNA length; Target GC% = 45; Oligonucleotide length = 20; Spacing length = 60-80. The 33 antisense *NEAT1* probes were divided into two pools: even and odd. RNA libraries of even- , odd- *NEAT1* pools and negative control (LacZ). Two pools of probes were used in order to exclude false-positive signals (unique to various probes). Only transcripts identified in both pools are considered to be true NEAT1-bound RNAs. RNA-Seq libraries were constructed using SMARTer® Stranded Total RNA v2 - Pico (Takara Bio USA, CA). ChIRP-seq was performed on Illumina HiSeq 2500 system (GeneWiz, NJ).

Immunocytochemistry. Mice were euthanized by deep anesthesia with compressed CO_2 gas followed by quick cervical dislocation before removing the brains**.** Brain tissue was cut in 12 µm serial sections. Primary antibody against *Qki*-5 isoform (1:1000 v/v, gift of Dr. Karen Artzt, Univ. of Texas at Austin and Dr. Monica Justice, Baylor College of Medicine) with secondary anti-rabbit AF488 conjugated antibodies (ThermoFisher, CA) were used. Sections stained with secondary antibodies alone were used as controls. DAPI counterstained sections were photographed using a Carl Zeiss AxioImager Z1 microscope and AxioVision Digital Image Processing System version 4.8.2.

Flow cytometry. Brain tissue disassociation, nuclei purification with sucrose gradient and antibody staining were carried out according to previously published protocol [15]. Primary antibodies: *Olig2*-FITC (1:50 v/v, EMD Millipore, MO) and *NeuN*-AlexaFluor 647 (1:1000 v/v, Abcam, MA) were used. Flow cytometry was performed on a BD Melody (BD Biosciences, CA). The data were analyzed by the FCS Express 6 software (De Novo Software, CA). Doublets

discrimination analysis was done based on signal processing (height vs. width). *Olig2*⁺ and *NeuN*⁺ cells were determined based on the FITC and AF674 fluorescence, correspondently.

Figures exemplifying the gating strategy:

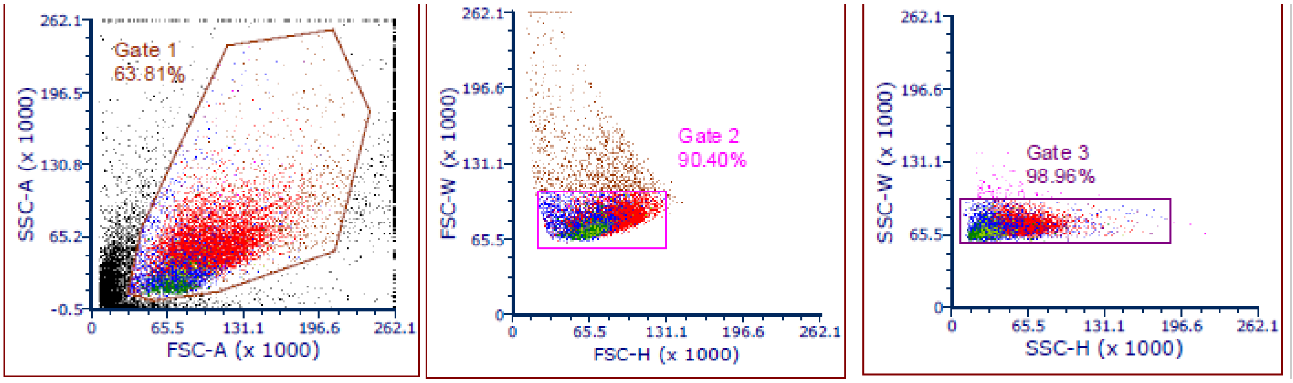

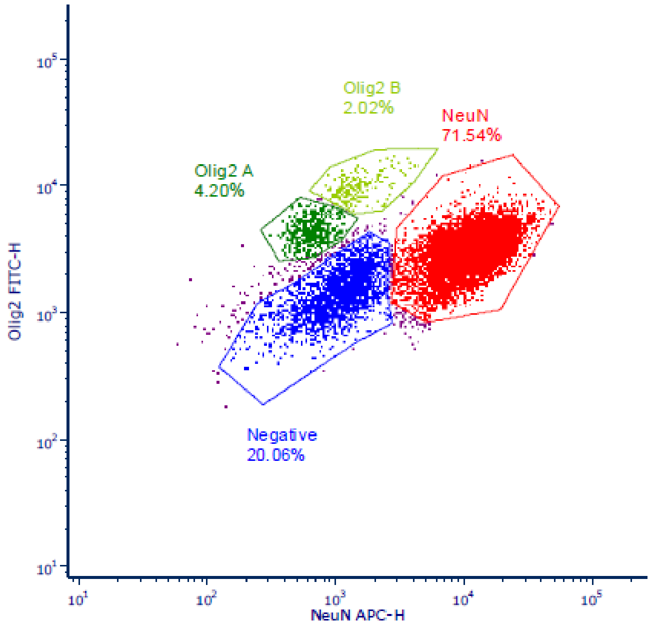

Reference List

1 Katsel, P., Davis, K. L., Gorman, J. M. & Haroutunian, V. Variations in differential gene expression patterns across multiple brain regions in schizophrenia. *Schizophr. Res.* **77**, 241-252, doi:10.1016/j.schres.2005.03.020 (2005).

2 Katsel, P. *et al.* Abnormal indices of cell cycle activity in schizophrenia and their potential association with oligodendrocytes. *Neuropsychopharmacology* **33**, 2993-3009, doi:10.1038/npp.2008.19 (2008).

3 De Preter, K., Barriot, R., Speleman, F., Vandesompele, J. & Moreau, Y. Positional gene enrichment analysis of gene sets for high-resolution identification of overrepresented chromosomal regions. *Nucleic Acids Res.* **36**, e43, doi:10.1093/nar/gkn114 (2008).

4 Katsel, P. & Haroutunian, V. in *Society for Neuroscience Abstracts* (2006).

5 Perry, T. L., Hansen, S. & Jones, K. Schizophrenia, tardive dyskinesia, and brain GABA. *Biol. Psychiatry* **25**, 200-206 (1989).

6 Davis, K. L. *et al.* White matter changes in schizophrenia: evidence for myelin-related dysfunction. *Arch. Gen. Psychiatry* **60**, 443-456, doi:10.1001/archpsyc.60.5.443 (2003).

7 Franklin, K. B. J. & Paxinos, G. *The Mouse Brain in Stereotaxic Coordinates*. (Academic Press, 1997).

8 Zeman, W. & Innes, J. R. M. *Craigie's neuroanatomy of the rat*. (Academic Press, 1963).

9 Fromer, M. *et al.* Gene expression elucidates functional impact of polygenic risk for schizophrenia. *Nat. Neurosci.*, doi:10.1038/nn.4399 (2016).

10 Robinson, M. D. & Oshlack, A. A scaling normalization method for differential expression analysis of RNA-seq data. *Genome Biol.* **11**, R25, doi:10.1186/gb-2010-11-3-r25 (2010).

11 Ritchie, M. E. *et al.* limma powers differential expression analyses for RNA-sequencing and microarray studies. *Nucleic Acids Res.* **43**, e47, doi:10.1093/nar/gkv007 (2015).

12 Zhang, B. & Horvath, S. A general framework for weighted gene co-expression network analysis. *Stat. Appl. Genet. Mol. Biol.* **4**, Article17, doi:10.2202/1544-6115.1128 (2005).

13 Ravasz, E., Somera, A. L., Mongru, D. A., Oltvai, Z. N. & Barabasi, A. L. Hierarchical organization of modularity in metabolic networks. *Science* **297**, 1551-1555, doi:10.1126/science.1073374 (2002).

14 Langfelder, P., Zhang, B. & Horvath, S. Defining clusters from a hierarchical cluster tree: the Dynamic Tree Cut package for R. *Bioinformatics* **24**, 719-720, doi:10.1093/bioinformatics/btm563 (2008).

15 Kozlenkov, A. *et al.* Substantial DNA methylation differences between two major neuronal subtypes in human brain. *Nucleic Acids Res.* **44**, 2593-2612, doi:10.1093/nar/gkv1304 (2016).
